# Polymorphisms of nucleotide excision repair genes associated with colorectal cancer risk: Meta-analysis and trial sequential analysis

**DOI:** 10.3389/fgene.2022.1009938

**Published:** 2022-10-31

**Authors:** Chuncheng Yi, Tiandong Li, Yajing Shen, Peng Wang, Liping Dai, Jianxiang Shi, Keyan Wang, Changqing Sun, Hua Ye

**Affiliations:** ^1^ College of Public Health, Zhengzhou University, Zhengzhou, China; ^2^ Henan Key Laboratory of Tumor Epidemiology and State Key Laboratory of Esophageal Cancer Prevention and Treatment, Zhengzhou University, Zhengzhou, China; ^3^ Henan Institute of Medical and Pharmaceutical Sciences, Zhengzhou University, Zhengzhou, China; ^4^ School of Nursing and Health, Zhengzhou University, Zhengzhou, China

**Keywords:** colorectal cancer, risk, single nucleotide polymorphisms, meta-analysis, nucleotide excision repair gene

## Abstract

**Background:** Reduced DNA repair capacity in nucleotide excision repair (NER) pathways owing to genetic variant may influence cancer susceptibility. According to published studies, variants of NER genes associations with colorectal cancer (CRC) risk were inconclusive. Thus, this meta-analysis aimed to explore the possible association. A trial sequence analysis (TSA) analysis was performed to control the risk of false positive or false negative.

**Methods:** PubMed, Web of Science, Embase, Cochrane Library, China National Knowledge Network (CNKI), Wanfang Database and Scientific and Technical Journal Database (VIP) were searched to identify relative studies until April 2022. The association was assessed by odds ratio (OR) in Allele, homozygous, heterozygous, dominant, recessive, and over-dominant models. In addition, Begg’s and Egger’s tests, sensitivity analysis, subgroup analysis and TSA analysis were performed.

**Results:** A total of 29 studies were eventually included in the meta-analysis, including 12,153 CRC patients and 14,168 controls. It showed that excision and repair cross complementary group 1 (*ERCC1*) rs11615 CC genotype decreased the risk of CRC, compared with TT genotype (CC vs. TT: OR = 0.816, 95% CI = 0.673–0.990, *p* = 0.039). For *ERCC1* rs3212986, the significant impact was detected on increased the risk of CRC in the allele (OR = 1.267, 95% CI = 1.027–1.562, *p* = 0.027), homozygous (OR = 1.805, 95% CI = 1.276–2.553, *p* = 0.001), dominant (OR = 1.214, 95% CI = 1.012–1.455, *p* = 0.037) and recessive (OR = 1.714, 95% CI = 1.225–2.399, *p* = 0.002) models, especially in the Asian population. The results revealed the association of *ERCC2* rs1799793 A allele with a higher risk of CRC (A vs. G: OR = 1.163, 95% CI = 1.021–1.325, *p* = 0.023). It also showed that *ERCC5* rs17655 increased CRC risk in the allele (OR = 1.104, 95% CI = 1.039–1.173, *p* = 0.001), homozygous (OR = 1.164, 95% CI = 1.018–1.329, *p* = 0.026), heterozygous (OR = 1.271, 95% CI = 1.018–1.329, *p* < 0.001), dominant (OR = 1.241, 95% CI = 1.135–1.358, *p* < 0.001) and over-dominant (OR = 0.828, 95% CI = 0.762–0.900, *p* < 0.001) models, especially among Asians.

**Conclusion:** This meta-analysis based on current evidence suggests that the significant association was observed between *ERCC1* rs11615, *ERCC1* rs3212986, *ERCC2* rs1799793, and *ERCC5* rs17655 and CRC susceptibility. However, given the limited sample size and the influence of genetic background, studies of a larger scale and well-designed are required to confirm the results.

## 1 Introduction

Globally, colorectal cancer (CRC) has the third highest incidence and second highest mortality rate, with a 5-year relative survival rate of 65% (Sung et al., 2021). By 2030, 2.2 million new cases and 1.1 million cancer deaths will occur, up 60% from today (Arnold et al., 2017). The mechanisms of CRC are multifactorial and multistage, influenced by hereditary, environmental factors, and their interaction (Khan et al., 2017). Smoking, excessive drinking and a diet heavy in red meat are all identified as potential risk factors of CRC (Baroudi and Benammar-Elgaaied, 2016). Besides, approximately 20%–30% of CRC patients have a family history of the disease, which demonstrates that genetic factors have been considered to be necessary in the development of CRC (Butterworth et al., 2006; Rasool et al., 2014).

DNA repair systems play a crucial role in preventing mutations or repairing DNA damage caused by environmental and genetic carcinogens (Sancar et al., 2004; Chatterjee and Walker, 2017). Nucleotide excision repair (NER) pathway is the first and most important step in any DNA repair process. It is a general mechanism for removing helical twisted DNA damage and structure from the genome (Marteijn et al., 2014). Its major types of damage repair involves radiation, chemotherapeutic agents and/or mutagens, such as cyclobutane pyrimidine dimer (CPD) and pyrimidine 6-4 (64 PP) caused by Ultraviolet B (UVB) radiation (Gillet and Schärer, 2006). There are two subpaths in NER. One is transcription-coupled NER(TC-NER) and the other is global genome NER(GG-NER). They are selective about which DNA strands are transcribed in the expressed genes. Many genes have been identified in the NER pathway, such as Xeroderma pigmentosum group C (*XPC*), *XPA*, excision and repair cross complementary group 1 (*ERCC1*), *ERCC2/XPD*, *ERCC4/XPF*, *ERCC5/XPG* (Hanawalt, 2002; Al-Shaheri et al., 2020).

Mutations in NER genes may result in a variety of phenotypes, range from an predisposition to cancer, to neurodevelopmental abnormalities defects associated with premature ageing (Marteijn et al., 2014). For example, *XPC* patients with defects in GG-NER have a 1,000-fold increased susceptibility to sun-induced skin cancer, as well as an increased risk of various internal tumors (DiGiovanna and Kraemer, 2012). Currently, the relationship between variants of NER gene and CRC risk has been reported in different populations. Some reports have shown that variants in NER genes lead to considerable differences in DNA repair ability between individuals, thus affecting their susceptibility to CRC (Dziki et al., 2017). Many studies have demonstrated the association between *ERCC2* rs13181 polymorphism and CRC among Asians (Yeh et al., 2007; Gil et al., 2012; Kabzinski et al., 2015), while others indicated no association (Jelonek et al., 2010; Wang et al., 2010). Paszkowska-Szczur et al. (2015) reported the association between single nucleotide polymorphisms (SNPs) of seven NER genes and CRC risk in the Polish population, which confirmed that variants in *XPC* rs2228000, *ERCC2* rs1799793 and rs238406 might be associated with CRC risk. *ERCC5* rs17655 variant related to increased CRC susceptibility in some case-control studies among Polish and Chinese populations (Gil et al., 2012; Su et al., 2019) but not others (Steck et al., 2014; Kabzinski et al., 2015). Due to the inconsistent and incomprehensive results of previous studies, this paper aims to systematically evaluate the association between SNPs in NER genes and the risk of CRC.

## 2 Materials and methods

This meta-analysis adhered to the Preferred Reporting Items for Systematic Reviews and Meta-Analyses (PRISMA) Statement (Page et al., 2021).

### 2.1 Retrieval strategy

The terms used in search strategy were “colorectal OR colonic OR rectal OR colon OR rectum,” “cancer OR carcinoma OR neoplasms OR tumor,” “Single Nucleotide Polymorphism OR Polymorphism OR SNP OR variant OR variation,” and “nucleotide excision repair OR NER OR DNA repair OR excision repair.” In addition, studies published as of 6 April 2022 were extracted from PubMed, Cochrane Library, Web of Science, Embase, China National Knowledge Network (CNKI), Wanfang Database, and Scientific and Technical Journal Database (VIP) to meet the search strategy, regardless of language restrictions. Detailed strategies are shown in Supplementary Information.

### 2.2 Inclusion criteria

Inclusion criteria for the study were as follows: 1) It was a case-control study; 2) The difference in SNP frequency between CRC patients and healthy controls was compared; 3) The studied SNPs were NER gene SNPs; 4) Cancer risk is the outcome; 5) Cases were serum samples from CRC patients who did not receive chemotherapy; 6) The control group included healthy individuals with different age, sex, country, and tumor stage and patients with non-malignant disease.

### 2.3 Data extraction

Two authors (Chuncheng Yi and Tiandong Li) independently extracted the literature. Data extracted from individual papers included: author, publication year, country, ethnicity, sample size, control type, gender composition, age at diagnosis, and details of target SNPs, including genotyping method, genotype frequency, and source from Hardy-Weinberg equilibrium (HWE).

### 2.4 Quality assessment

The quality of the studies that met the inclusion criteria was assessed. Two reviewers assessed the methodological quality of each study against the Newcastle-Ottawa scale (NOS) for case-control study (Stang, 2010). Scores greater than six are considered high quality, and the total score was nine.

### 2.5 Statistical analysis

A 95% confidence interval (95% CI) and odds ratio (OR) were utilized to determine the association between SNPs in NER gene and risk of CRC, and OR was analyzed using the Z test. Six different genetic models were used to test the association, including allelic, homozygous, heterozygous, dominant, recessive, and over-dominant models. Heterogeneity was assessed by Q test and I^2^ tests. I^2^ < 25% was considered to be of low heterogeneity, 25%–50% to be of moderate heterogeneity and 50% was considered a high level of heterogeneity. *p* < 0.05 or I^2^ > 50% was considered significant for heterogeneity, and a Dersimonian-Laird random effect model was applied if heterogeneity was not detected; otherwise, a Mantel-Haenszel fixed effect model was applied. Subgroup analyses were performed based on ethnicity and source of control to explore potential heterogeneity. A sensitivity analysis was performed to assess the stability of the results by excluding each study individually. The funnel plot evaluated whether publication bias was present (both Begg’s and Egger’s tests). For all statistical analyses, Stata version 14.0 software was used (StataCorp LP, College Station, TX, United States).

### 2.6 Trial sequential analysis

To control the risk of false positive or false negative, a trial sequential analysis (TSA) method was performed. If the curve exceeds the traditional boundary but does not cross the TSA boundary, the prompt may make a false positive error; If the curve intersects TSA boundary, the meta-analysis results are stable, even if required information size (RIS), which is the target value needed to make a reliable statistical inference, is not reached; If the curve does not intersect with both the traditional and TSA bounds, no positive or negative conclusion can be drawn; If the curve intersects an invalid line, the hint is meaningless. We performed TSA with a type I error risk of 5%, a power of 90% and a relative risk reduction of 15%, using TSA version 0.9.5.10 software (Copenhagen Trial Unit, Copenhagen, Denmark; http://www.ctu.dk/tsa).

## 3 Results

### 3.1 Description of included studies

**FIGURE 1:**
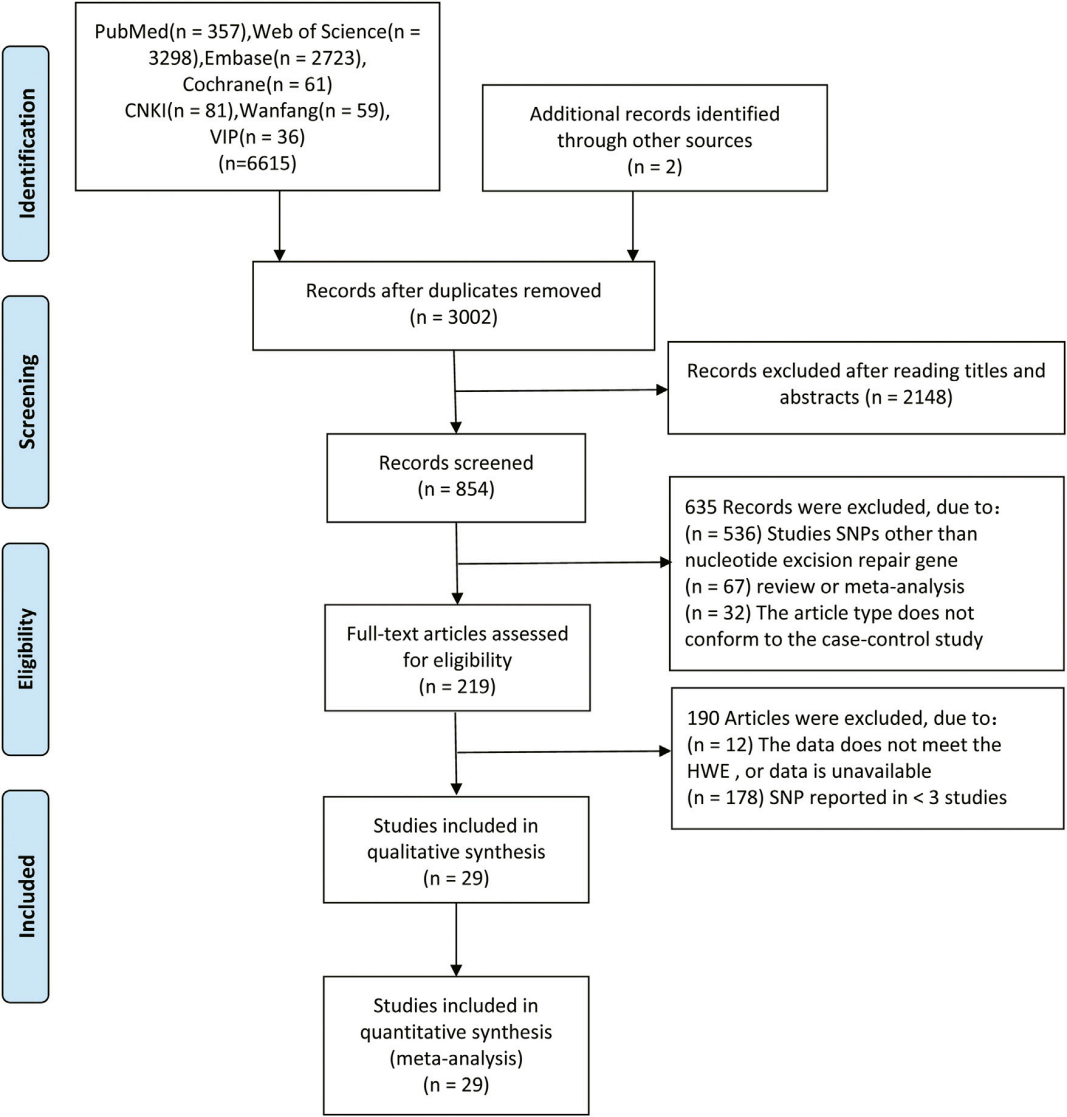
Flow diagram of the literature search in this meta-analysis.

The flow chart of this study is shown in Figure 1. In total, 6,617 relevant articles were initially retrieved in this study including two articles obtained from cited references. Among them, 3,615 duplicated articles, 2,148 articles with the irrelevant subject after reading titles and abstracts, 536 articles with SNPs other than NER gene, 67 review or meta-analysis articles and 32 not case-control studies were excluded. After evaluating the remaining 217 articles, 12 studies with unavailable data or data do not meet the HWE, and 178 studies reporting SNP in less than three studies. Therefore, a total of 29 studies (Moreno et al., 2006; Skjelbred et al., 2006; Stern et al., 2006; Yeh et al., 2007; Pardini et al., 2008; Sliwinski et al., 2009; Wang et al., 2009; Engin et al., 2010; Jelonek et al., 2010; Wang et al., 2010; Canbay et al., 2011; Gil et al., 2012; Liu et al., 2012; Du et al., 2014; Hou et al., 2014; Ni et al., 2014; Steck et al., 2014; Gómez-Díaz et al., 2015; Kabzinski et al., 2015; Paszkowska-Szczur et al., 2015; Sun et al., 2015; Yang et al., 2015; Yueh et al., 2017; Zhang et al., 2017; Zhang et al., 2018; Jin et al., 2019; Su et al., 2019; Balkan et al., 2020; Li et al., 2020) were included with 12,153 CRC patients and 14,168 controls. The characteristics of the studies included in the meta-analysis were showed in Table 1. The genotype distributions of eight SNPs in the five genes in this study agreed with the Hardy-Weinberg equilibrium (*p* > 0.05). The result of quality assessment is shown in Supplementary Table S1 and all included studies were of high quality. Table 2 shows the genotype frequency distributions of included studies.

**TABLE 1:** Characteristics of studies included for meta-analysis.

First author, year of publication	Country	Ethnicity	Studied SNPs	Genotyping method	Sample size (case/control)	P_HWE_ in controls	Source of controls
Mariana C Stern 2006	United States	Mixed	*ERCC2* rs13181	PCR-RFLP	855/901	0.18	HB
Victor Moreno 2006	Spain	Caucasian	*ERCC1* rs11615, *ERCC1* rs3212986, *ERCC2* rs13181,	APEX	377/329	0.05/0.31/0.92/3.71/0.53	HB
			*ERCC2* rs1799793, *ERCC4* rs1800067				
C.Furu Skjelbred 2006	Norway	Caucasian	*ERCC2* rs13181	TaqMan	157/399	0.48	HB
Chih-Ching Yeh 2007	Taiwan	Asian	*ERCC2* rs13181	PCR-RFLP	727/736	0.87	HB
B Pardini 2008	Czech	Caucasian	*ERCC5* rs17655	PCR-RFLP	532/532	0.21	HB
Tomasz Sliwinski 2009	Poland	Caucasian	*ERCC2* rs13181	PCR-RFLP	100/100	0.21	HB
Wang LiLi 2009	China	Asian	*ERCC2* rs13181	PCR-RFLP	170/200	0.38	HB
K Jelonek 2010	Poland	Caucasian	*ERCC2* rs13181, *ERCC2* rs1799793	PCR-RFLP	153/153	0.82/0.06	HB
Ayse Basak Engin 2010	Turkey	Asian	*XPC* rs2228001	PCR-RFLP	110/116	0.64	HB
Jingwen Wang 2010	India	Asian	*ERCC2* rs13181	PCR-RFLP	302/291	0.13	PB
Emel Canbay 2011	Turkey	Asian	*ERCC2* rs13181, *ERCC5* rs17655	PCR-RFLP	79/247	0.92/0.35	PB
Duo Liu 2012	China	Asian	*ERCC5* rs17655, *XPC* rs2228001	PCR-RFLP	1,028/1,085	0.10/0.74	HB
Justyna Gil 2012	Poland	Caucasian	*ERCC2* rs13181, *ERCC2* rs1799793, *ERCC4* rs1800067,	PCR-RELP	133/100	0.48/0.08/0.41/0.62/0.80	HB
			*ERCC5* rs17655, *XPC* rs2228001				
Susan E Steck 2014 group 1	United States	Caucasian	*ERCC2* rs13181, *ERCC4* rs1800067, *ERCC5* rs17655,	MassARRAY	349/611	0.44/0.05/0.37/0.70/0.85	PB
			*XPC* rs2228001, *XPC* rs2228000				
Susan E Steck 2014 group 2	United States	African	*ERCC2* rs13181, *ERCC2* rs1799793, *ERCC4* rs1800067,	MassARRAY	294/437	0.83/0.81/0.65/0.52/0.74/0.16	PB
			*ERCC5* rs17655, *XPC* rs2228001, *XPC* rs2228000				
Min Ni 2014	China	Asian	*ERCC1* rs11615, *ERCC1* rs3212986, *ERCC2* rs13181,	TaqMan	213/240	0.31/0.43/0.57/0.06	PB
			*ERCC2* rs1799793				
Ruizhi Hou 2014	China	Asian	*ERCC1* rs11615, *ERCC1* rs3212986	MassARRAY	204/204	0.51/0.80	PB
Haina Du 2014	China	Asian	*ERCC5* rs17655	TaqMan	878/884	0.62	PB
Kang Sun 2015	China	Asian	*XPC* rs2228000	PCR-RELP	890/910	4.98	PB
K.P-Szczur 2015	Poland	Caucasian	*ERCC2* rs13181, *XPC* rs2228001, *XPC* rs2228000	TaqMan	758/1841	0.31/0.71/0.09	PB
H. Yang 2015	China	Asian	*ERCC1* rs11615	PCR-RFLP	279/316	0.11	HB
B. GÓMEZ-DÍAZ 2015	Mexico	Mixed	*ERCC1* rs11615, *ERCC2* rs13181, *ERCC2* rs1799793	TaqMan	108/119	0.96/0.77/1.45	HB
Jacek Kabzinski 2015	Poland	Caucasian	*ERCC2* rs13181, *ERCC2* rs1799793, *ERCC5* rs17655	TaqMan	235/240	1.35/1.45/7.79	HB
Sha Zhang 2017	China	Asian	*ERCC2* rs13181	TaqMan	338/315	0.12	PB
Te-Cheng Yueh 2017	Taiwan	Asian	*ERCC1* rs11615	PCR	362/362	0.05	PB
Qianye Zhang 2018	China	Asian	*ERCC1* rs3212986	TaqMan	200/200	0.62	PB
Dexi Jin 2019	China	Asian	*ERCC2* rs13181	PCR-RFLP	225/200	0.20	PB
Jinsong Su 2019	China	Asian	*ERCC5* rs17655	TaqMan	1,019/1,036	0.85	HB
Eda Balkan 2020	Turkey	Asian	*ERCC2* rs13181	LightSNiP	40/40	0.22	HB
Yan-Ke Li 2020	China	Asian	*ERCC1* rs11615	PCR	1,038/1,024	0.92	HB

Note: 1. P_HWE_ represents the *p* value of hardy-Weinberg balance law test. 2. HB, hospital-based; PB, population-based.

**TABLE 2:** Results of meta-analysis of the selected SNPs.

Genetic model	Sample size	Heterogeneity		Model	OR (95%CI)	Genetic model	Sample size	Heterogeneity		Model	OR (95%CI)
		Case/control	I^2^ (%)				P	Case/control	I^2^ (%)	P	
*ERCC1* rs11615	2,538/2,566					*ERCC4* rs1800067	1,110/1,449				
C vs. T		44.20	0.096	Fixed	0.925 (0.847–1.010)	A vs. G		0.00	0.668	Fixed	1.026 (0.821–1.281)
CC vs. TT*		38.50	0.135	Fixed	0.816 (0.673–0.990)	AA vs. GG		22.60	0.275	Fixed	0.914 (0.380–2.201)
CT vs. TT		8.70	0.362	Fixed	0.864 (0.711–1.050)	AG vs. GG		6.30	0.362	Fixed	1.050 (0.822–1.343)
CC+CT vs. TT		33.00	0.176	Fixed	0.834 (0.695–1.000)	AA+AG vs. GG		0.00	0.517	Fixed	1.040 (0.818–1.322)
CC vs. CT+TT		0.00	0.452	Fixed	0.941 (0.838–1.056)	AA vs. AG+GG		25.30	0.262	Fixed	0.898 (0.374–2.155)
CC+TT vs. CT		0.00	0.963	Fixed	1.013 (0.901–1.139)	AA+GG vs. AG		10.00	0.343	Fixed	0.950 (0.744–1.215)
*ERCC1* rs3212986	2,067/2,119					*ERCC5* rs17655	5,648/6,347				
A vs. C*		52.40	0.098	Random	1.267 (1.027–1.562)	C vs. G*		0.00	0.887	Fixed	1.104 (1.039–1.173)
AA vs. CC*		21.50	0.281	Fixed	1.805 (1.276–2.553)	CC vs. GG*		0.00	0.697	Fixed	1.164 (1.018–1.329)
AC vs. CC		31.90	0.221	Fixed	1.121 (0.925–1.358)	CG vs. GG*		31.90	0.163	Fixed	1.271 (1.157–1.396)
AA+AC vs. CC*		48.70	0.119	Fixed	1.214 (1.012–1.455)	CC+CG vs. GG*		10.60	0.347	Fixed	1.241 (1.135–1.358)
AA vs. AC+CC*		0.00	0.480	Fixed	1.714 (1.225–2.399)	CC vs. CG+GG		0.00	0.602	Fixed	0.998 (0.889–1.119)
AA+CC vs. AC		0.00	0.401	Fixed	0.969 (0.804–1.167)	CC+GG vs. CG*		38.70	0.110	Fixed	0.828 (0.762–0.900)
*ERCC2* rs13181	5,585/7,470					*XPC* rs2228001	2,672/4,190				
C vs. A		79.80	<0.001	Random	1.060 (0.999–1.126)	T vs. G		55.90	0.045	Random	1.029 (0.900–1.177)
CC vs. AA		73.50	<0.001	Random	1.177 (0.854–1.622)	TT vs. GG		33.50	0.185	Fixed	1.063 (0.894–1.265)
CA vs. AA		34.50	0.071	Fixed	1.026 (0.942–1.118)	TG vs. GG		60.50	0.027	Random	1.220 (0.902–1.651)
CC+CA vs. AA		30.60	0.102	Fixed	1.044 (0.962–1.132)	TT+TG vs. GG		47.90	0.087	Fixed	1.117 (0.952–1.310)
CC vs. CA+AA		84.20	<0.001	Random	1.160 (0.809–1.662)	TT vs. TG+GG		71.30	0.004	Random	0.977 (0.771–1.238)
CC+AA vs. CA		82.00	<0.001	Random	1.042 (0.850–1.278)	TT+GG vs. TG		76.80	0.001	Random	0.908 (0.706–1.167)
*ERCC2* rs1799793	2,243/3,430					*XPC* rs2228000	2,291/3,799				
A vs. G		75.20	<0.001	Random	1.229 (0.988–1.528)	A vs. G		96.40	<0.001	Random	1.019 (0.614–1.689)
AA vs. GG		80.50	<0.001	Random	1.785 (0.989–3.221)	AA vs. GG		95.80	<0.001	Random	1.111 (0.310–3.984)
AG vs. GG		0.00	0.849	Fixed	1.088 (0.945–1.253)	AG vs. GG		89.30	<0.001	Random	0.944 (0.635–1.405)
AA+AG vs. GG*		35.20	0.148	Fixed	1.312 (1.153–1.494)	AA+AG vs. GG		94.20	<0.001	Random	0.972 (0.580–1.627)
AA vs. AG+GG*		80.50	<0.001	Random	1.783 (1.031–3.081)	AA vs. AG+GG		95.00	<0.001	Random	1.151 (0.374–3.545)
AA+GG vs. AG*		0.00	0.488	Fixed	1.141 (1.001–1.300)	AA+GG vs. AG		76.40	0.005	Random	1.102 (0.851–1.427)

Note: 1. Random stands for Random effect model, Fixed stands for Fixed effect model. When P > was 0.05 and I^2^ < 50% in the heterogeneity test, heterogeneity was considered to be small, the fixed effect model was used to combine the results, conversely, it is considered that the heterogeneity is large and the results are combined by the random effect model. 2. All the gene models with standard * were meaningful models for meta-analysis.

### 3.2 Quantitative analysis

Upon analysis of these eight NER-SNPs, we found that four of them were significantly associated with CRC risk. Detailed results are shown in Supplementary Table S2.

#### 3.2.1 Polymorphisms in *ERCC1* and colorectal cancer risk

**FIGURE 2:**
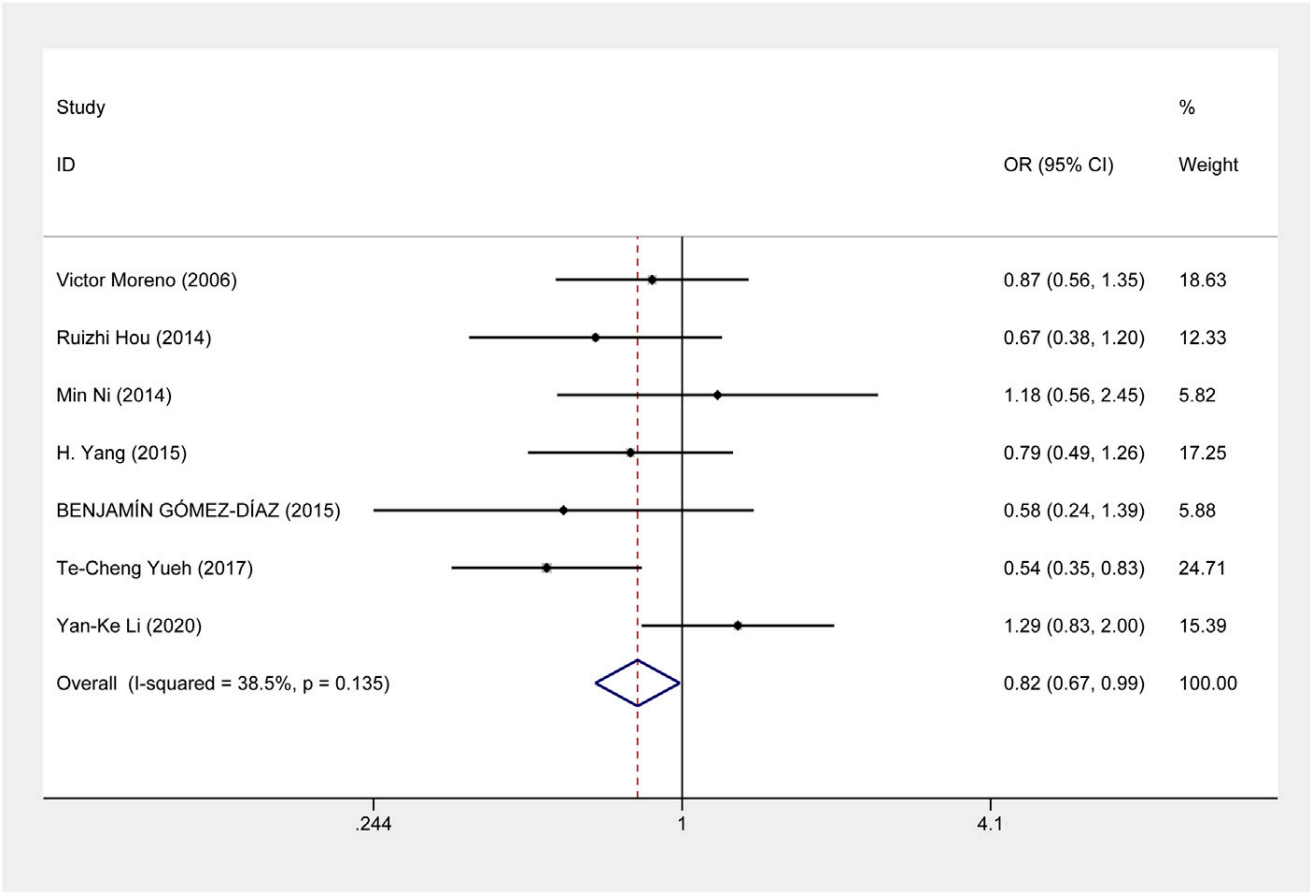
Forest plot related to *ERCC1* rs11615 and risk of CRC in the homozygous model.

**FIGURE 3:**
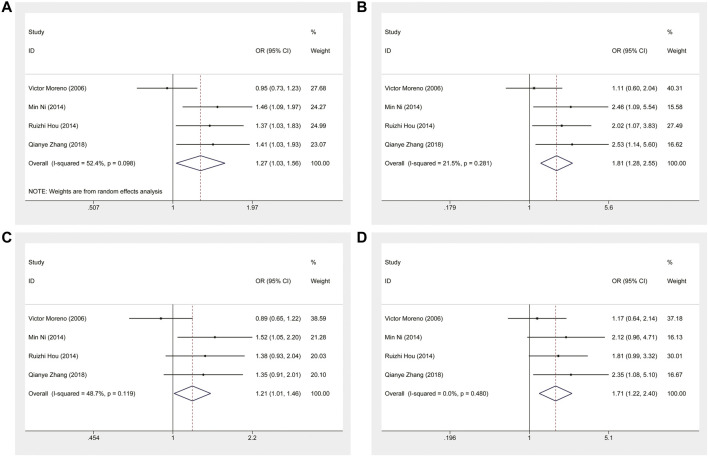
Forest plot related to *ERCC1* rs3212986 and risk of CRC. **(A)** Allele model **(B)** Homozygous model **(C)** Dominant model **(D)** Recessive model.

This meta-analysis showed that *ERCC1* rs11615 homozygous CC genotype, but not the heterozygous CT genotype, decreased the risk of CRC, compared with the wild-type TT genotype (CC vs. TT: OR = 0.816, 95% CI = 0.673–0.990, *p* = 0.039; CT vs. TT: OR = 0.864, 95% CI = 0.711–1.050, *p* = 0.141) (Figure 2), however, this association was not shown in subgroup analysis of ethnicity and source of control. For *ERCC1* rs3212986, the significant impact was detected on increased the risk of CRC under allele, homozygous, dominant and recessive models (A vs. C: OR = 1.267, 95% CI = 1.027–1.562, *p* = 0.027; AA vs. CC: OR = 1.805, 95% CI = 1.276–2.553, *p* = 0.001; AA+AC vs. CC: OR = 1.214, 95% CI = 1.012–1.455, *p* = 0.037; AA vs. AC+CC: OR = 1.714, 95% CI = 1.225–2.399, *p* = 0.002) (Figure 3). The stratified analysis by ethnicity and source of control revealed a significantly higher CRC risk was found for the Asian population and the population-based control subgroup (Supplementary Figure S1).

#### 3.2.2 Polymorphisms in *ERCC2/XPD* and colorectal cancer risk

**FIGURE 4:**
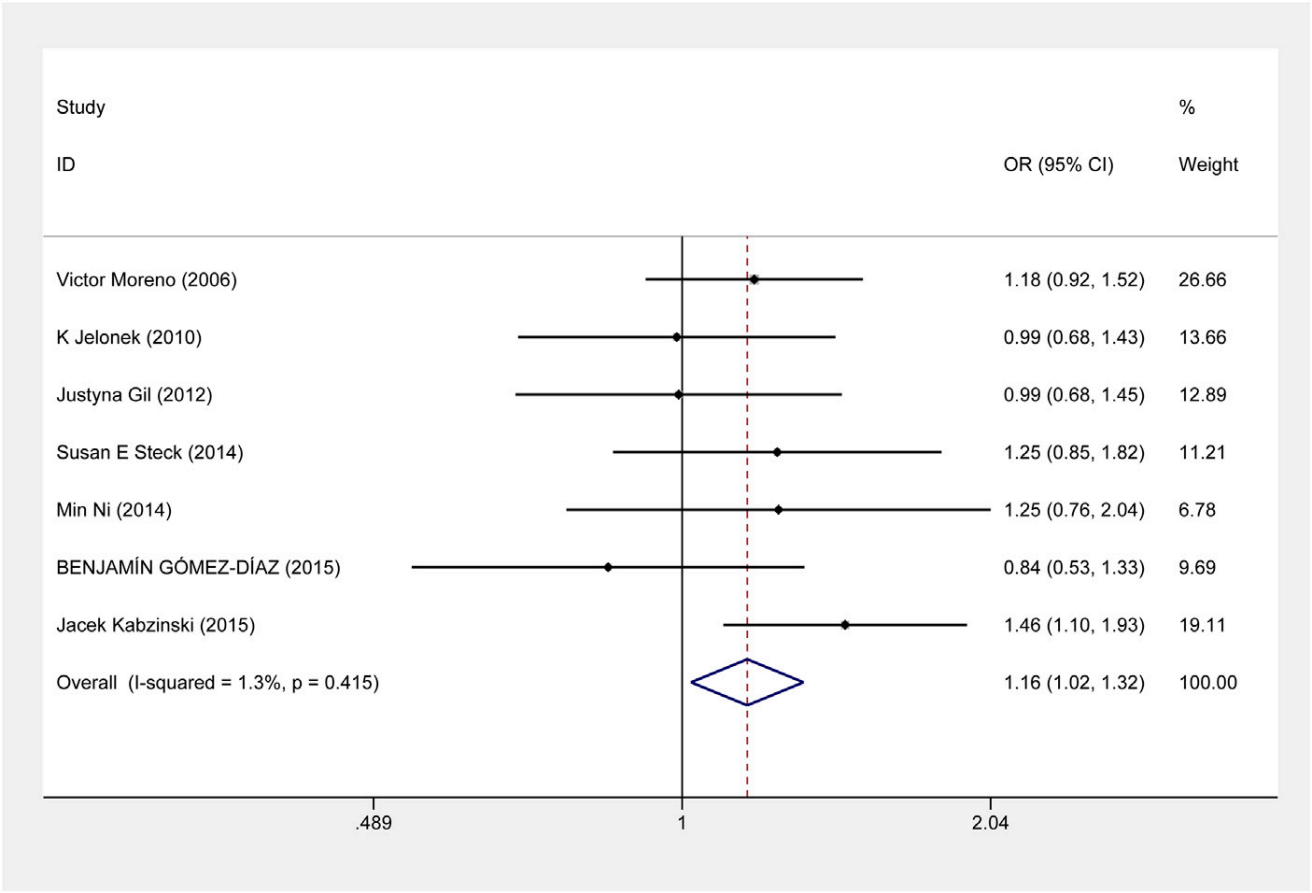
Forest plot related to *ERCC2* rs1799793 and risk of CRC in the allele model.

For *ERCC2* rs1799793, our meta-analysis showed that compared with G allele, A allele was associated with an increased risk of CRC (A vs. G: OR = 1.163, 95% CI = 1.021–1.325, *p* = 0.023) (Figure 4). And the association was also shown in the Caucasian population (Supplementary Figure S2). About the risk of CRC and *ERCC2* rs13181, there was no significant evidence of an association between them when all eligible studies were included under all genetic models. However, the stratified analysis by ethnicity revealed that *ERCC2* rs13181 CC/CA genotype was associated with a significantly increased risk of CRC, compared with AA genotype among the Asian population. (CC+CA vs. AA: OR = 1.205, 95% CI = 1.032–1.408, *p* = 0.018) (Supplementary Figure S3).

#### 3.2.3 Polymorphism in *ERCC5*/*XPG* and colorectal cancer risk

**FIGURE 5:**
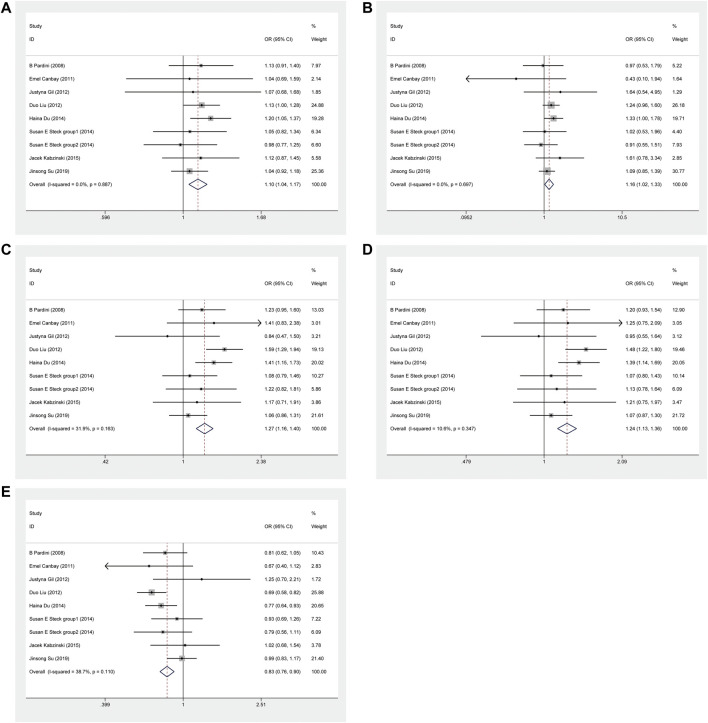
Forest plot related to *ERCC5* rs17655 and risk of CRC. **(A)** Allele model **(B)** Homozygous model **(C)** Heterozygous model **(D)** Dominant model **(E)** Over-dominant model.

The results demonstrated that significant associations between *ERCC5* rs17655 and CRC risk were noted under five genetic models. The correlation results under allele model was OR = 1.104(C vs. G: 95% CI = 1.039–1.173, *p* = 0.001), homozygous model was OR = 1.164(CC vs. GG: 95% CI = 1.018–1.329, *p* = 0.026), heterozygous model was OR = 1.271(CG vs. GG: 95% CI = 1.018–1.329, *p* < 0.001), dominant model was OR = 1.241(CC+CG vs. GG: 95% CI = 1.135–1.358, *p* < 0.001) and over-dominant model was OR = 0.828(CC+GG vs. CG: 95% CI = 0.762–0.900, *p* < 0.001) (Figure 5). Moreover, these significant associations exist in the Asian population under five genetic models and in the population-based control and hospital-based control subgroup under four models except the homozygous model (Supplementary Figure S4).

#### 3.2.4 Polymorphisms in *ERCC4*/*XPF* and *XPC* and colorectal cancer risk

No significant association was observed between *ERCC4* rs1800067 variant and CRC susceptibility in the six genetic models, nor in the stratified analysis. For *XPC* rs2228001 and rs2228000 variants, although no significant associations exist in the overall meta-analysis, ethnic subgroup analysis showed that significant correlations were noted in reducing or increasing the risk of CRC in Asian, Caucasian and African populations under different models. The detailed results are shown in Supplementary Figures S5, S6.

### 3.3 Heterogeneity and sensitivity analysis

Under the allelic model, *ERCC1* rs3212986 had high heterogeneity (I2 = 52.4%, *p* = 0.098). We then assessed the source of heterogeneity by ethnicity and control source. The results showed that heterogeneity among the Asian and population-based control subgroups decreased. Then, sensitivity analyses were performed to assess the stability of the results by deleting each study in sequence. The results showed that none of the studies significantly changed the combined OR, indicating that the combined OR of this variant was robust. Heterogeneity was low for the remaining meaningful SNPs, and the sensitivity analysis results were robust. Supplementary Figures S7–S10 for details.

### 3.4 Publication bias

Begg’s funnel plot and Egger’s regression test were performed to estimate publication bias. The results showed no significant asymmetry of funnel plot shape (Supplementary Figures S7–S10), and Begg’s and Egger’s test also showed that the evaluation of publication bias had no statistical significance.

### 3.5 Trial sequential analysis

**FIGURE 6:**
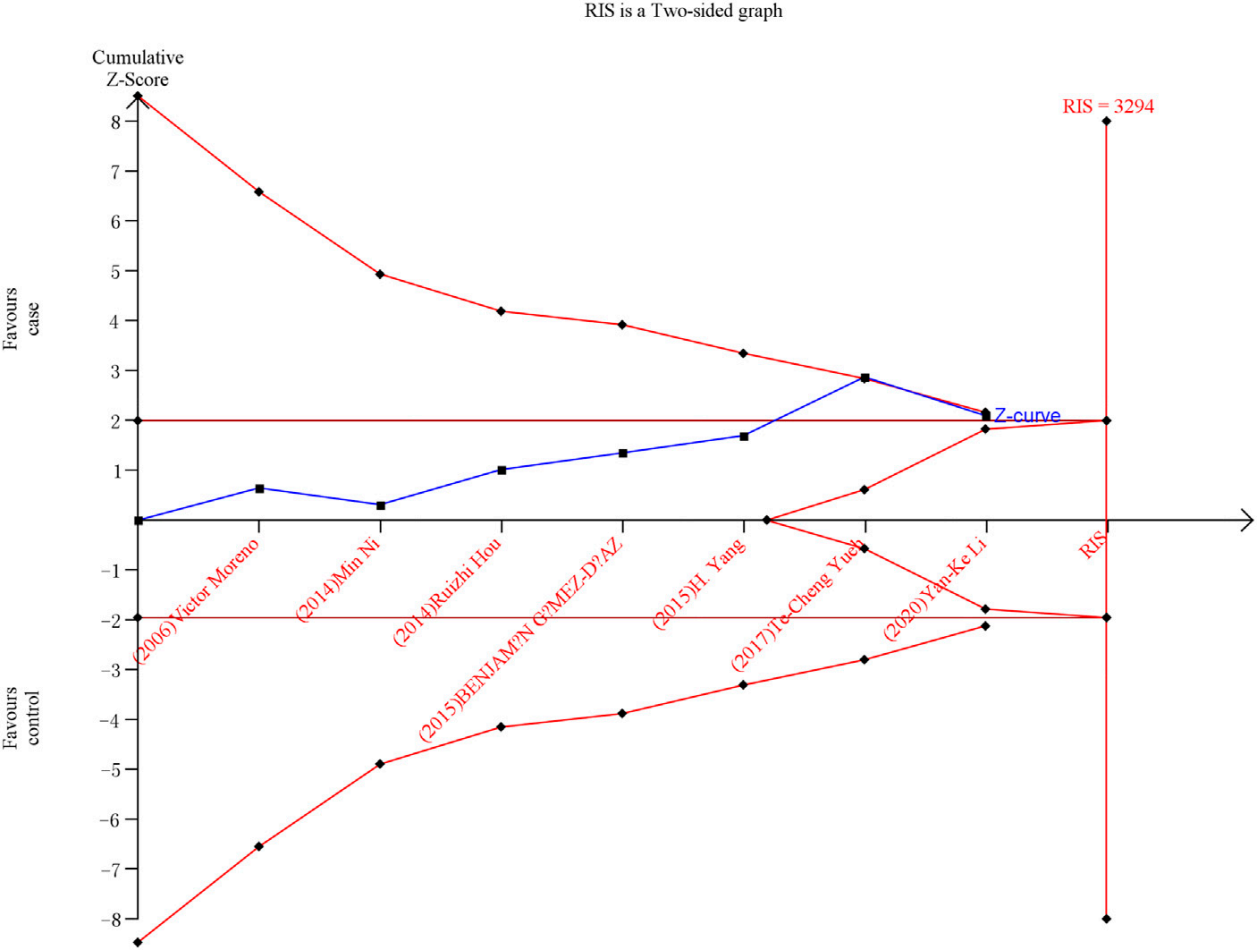
TSA for *ERCC1* rs11615 polymorphism and CRC risk in the homozygous model.

**FIGURE 7:**
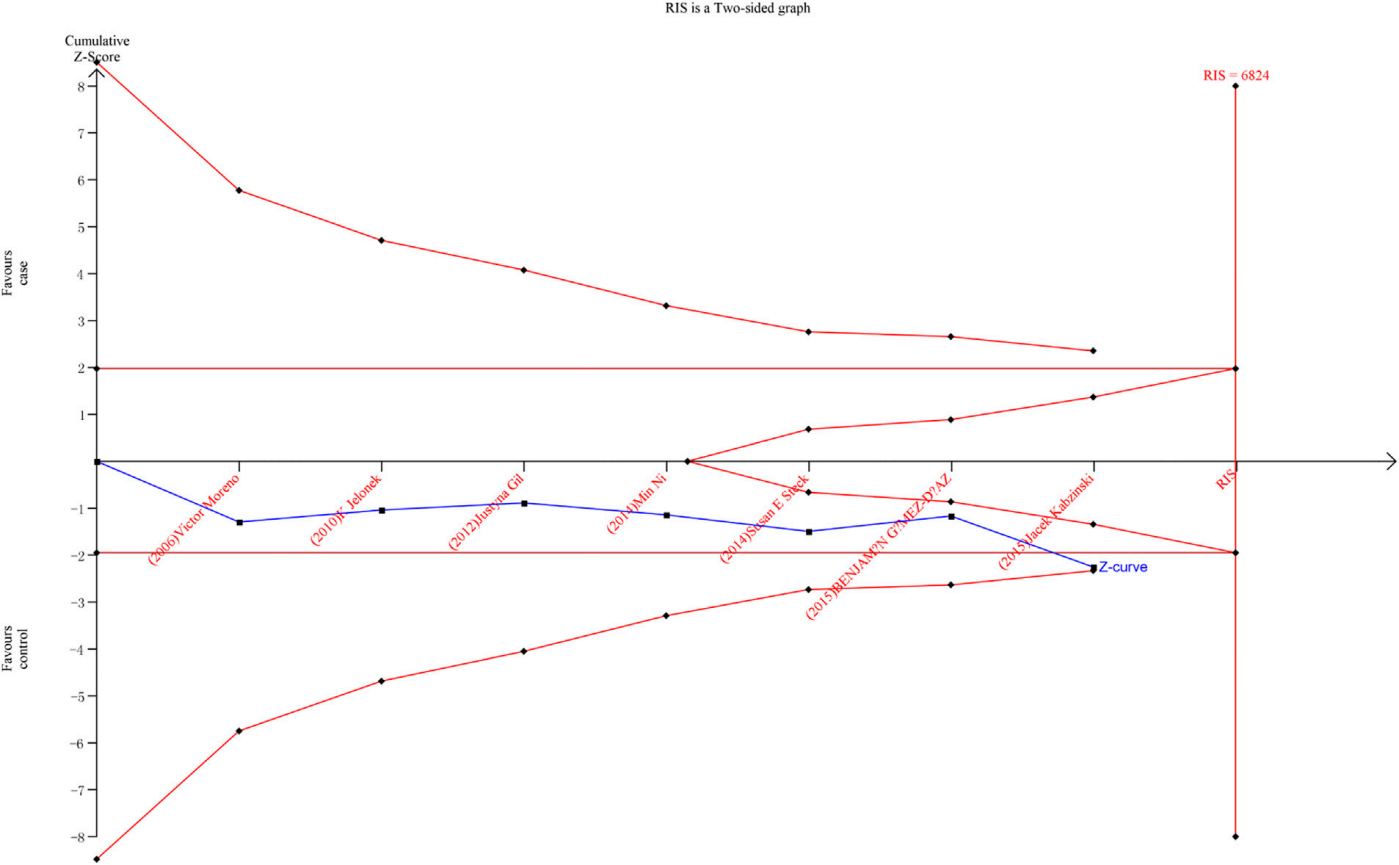
TSA for *ERCC2* rs1799793 polymorphism and CRC risk in the allele model.

**FIGURE 8:**
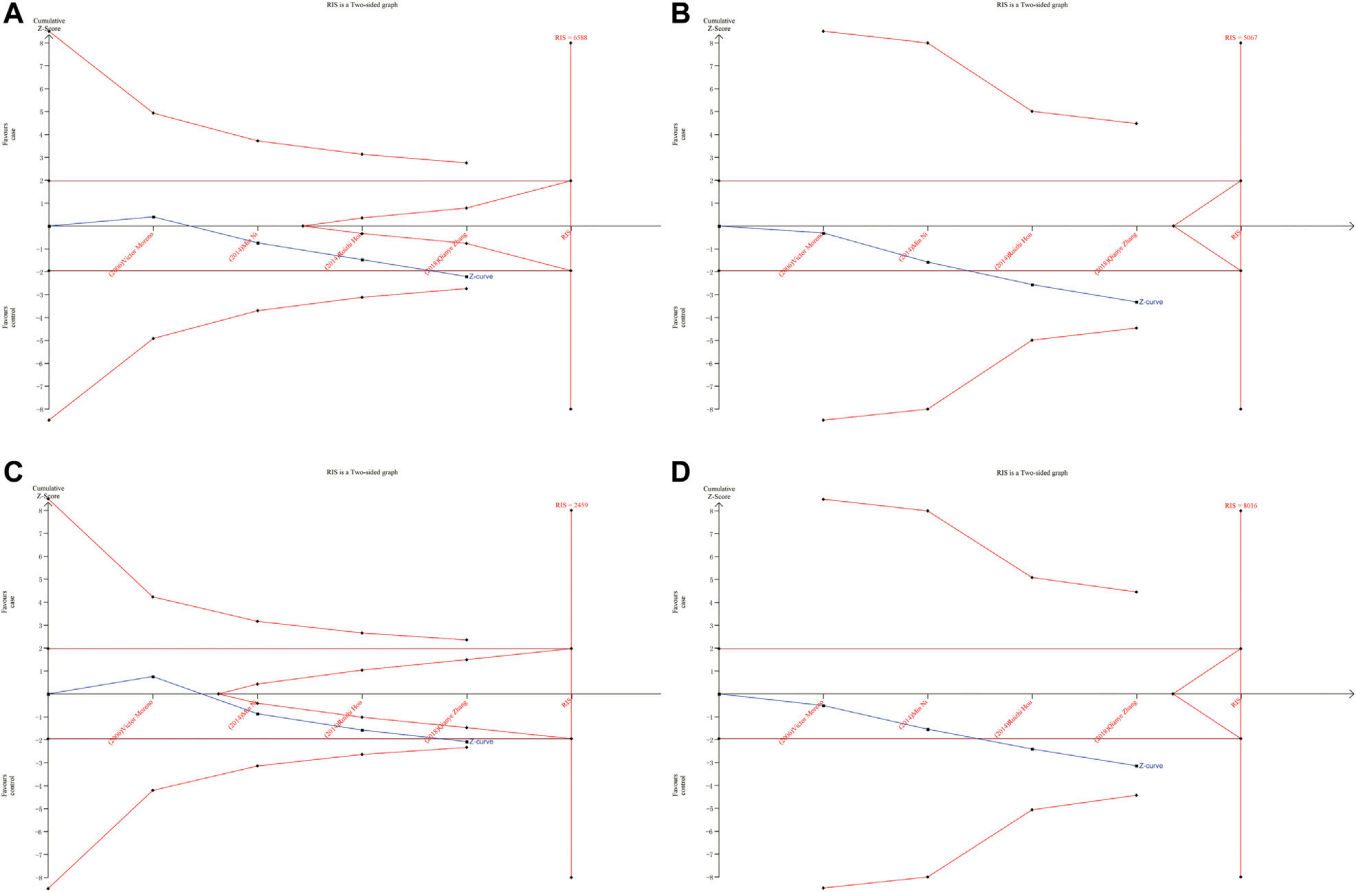
TSA for *ERCC1* rs3212986 polymorphism and CRC risk. **(A)** Allele model **(B)** Homozygous model **(C)** Dominant model **(D)** Recessive model.

**FIGURE 9:**
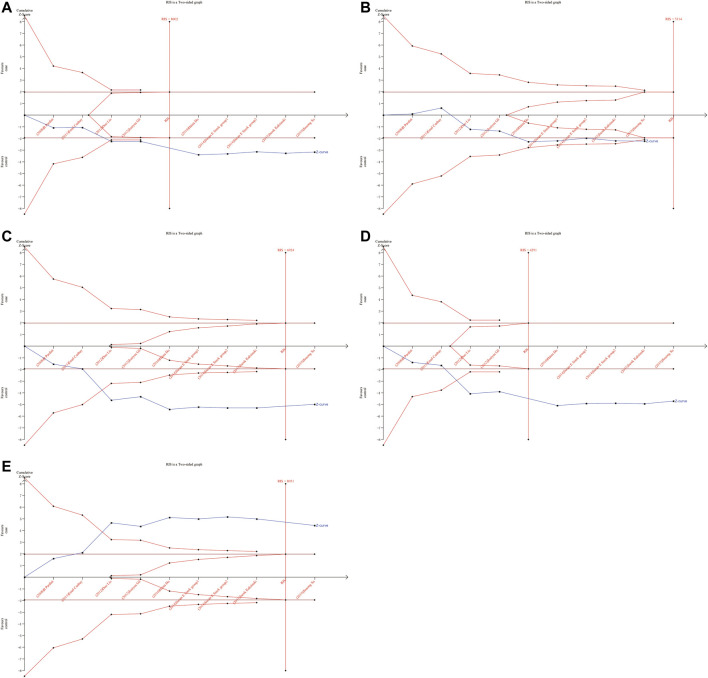
TSA for *ERCC5* rs17655 polymorphism and CRC risk. **(A)** Allele model **(B)** Homozygous model **(C)** Heterozygous model **(D)** Dominant model **(E)** Over-dominant model.

As shown in Figures 6, 7, although the accumulated information has not reached RIS, the Z curve has crossed the traditional boundary and TSA boundary. The cumulative evidence was sufficient to the association between *ERCC1* rs11615 variant and CRC risk under homozygous model, similar to *ERCC2* rs1799793. For *ERCC1* rs3212986 variant in the four genetic models, the Z curve only crossed the traditional boundary, and the sample size did not reach RIS, indicating that there was a high possibility of false positives in the results. Therefore, more subsequent studies are needed for further verification to make this association valid (Figure 8). For *ERCC5* rs17655 variant (Figure 9), its Z curve crossed the traditional boundary and TSA boundary, and the sample size reached RIS, demonstrating that the results were robust.

## 4 Discussion

The NER repair pathway is ubiquitous in all living organisms. It eliminates barriers to replication and transcription, as well as structures that may affect DNA stability or integrity (Spivak, 2015). NER repairs a wide range of lesions. Phenotypes of cancer susceptibility observed in GG-NER disorders. The deficiency of TC-NER impairs cell function and induces premature cell death, thus accelerating aging (Marteijn et al., 2014). Therefore, the mechanism of NER pathway and its impact on CRC susceptibility is an important area of research. Previous studies have shown that the expression changes of four NER genes in CRC are significantly different (Gómez-Díaz et al., 2015). In recent years, several epidemiological studies have described associations between the risk of CRC and SNPs in the NER repair pathway (Jelonek et al., 2010; Slyskova et al., 2012), while the results were inconsistent and incomprehensive.

We performed a meta-analysis to comprehensively discover the association between variants in NER gene and CRC susceptibility. After rigorous screening, a total of 29 case-control studies were eligible, including eight SNPs in the NER repair pathway. Our results showed that *ERCC1* rs11615, *ERCC1* rs3212986, *ERCC2* rs1799793, and *ERCC5* rs17655 variants were significantly correlated with CRC susceptibility. No significant impact of *ERCC2* rs13181 variant, *ERCC4* rs1800067 variant, *XPC* rs2228001 and *XPC* rs2228000 variants was detected on CRC risk.

*ERCC1* enzyme is part of the NER system. *ERCC1* protein is essential for maintaining genetic stability. Deletion of *ERCC1* protein is associated with cancer susceptibility and has been reported as a biomarker for platinum resistance in colorectal and gastric cancer patients (Kwon et al., 2007). Previous meta-analyses showed that *ERCC1* rs11615 variant was not associated with CRC risk (Chen et al., 2017). We added two more published studies to this basis, which showed that *ERCC1* rs11615 CC genotype was associated with a reduced occurrence of CRC, but this significance was not shown in further stratified analysis. TSA analysis showed that with the increase in sample size, the overall results tend to be meaningful. Although the cumulative sample size does not reach RIS, the Z curve has intersected with the boundary value, indicating that the current results are highly reliable and the possibility of false positives is not significant. For the *ERCC1* rs3212986 variant, our results showed AA genotype was associated with an increased risk of CRC. Further stratified analysis showed that the association persisted in the population-based control and Asian population subgroups. This is consistent with the results of previous studies by Chen et al. (2017). However, TSA analysis showed that Z curve only crossed the traditional boundary, and the total sample size did not reach RIS, indicating that the results had a large possibility of false positive, which requires further verification by follow-up studies to make the association valid.

*ERCC2* gene is an essential component of NER, an ATP-dependent 5′-3′ helices, and mutations at different sites of the *ERCC2* gene lead to repair and transcriptional defects (Spitz et al., 2001). Many studies have been performed to investigate the association of *ERCC2* variants with CRC risk with mixed results, in which the most informative variants are rs1799793 and rs13181 (Lunn et al., 2000). A previous meta-analysis suggested that rs1799793 and rs13181 polymorphisms may not be associated with the development of CRC (Zhang et al., 2011). As for rs13181, our results were consistent with the above findings. However, further subgroup analysis of the association between rs13181 variant and CRC revealed a significantly increased risk in the Asian population under the dominant model. Moreover, A allele was associated with an increased risk of CRC in rs1799793, which persisted in the Caucasian population. This inconsistency may be due to the relatively small sample size of each study and the possible weak effect of polymorphism on CRC risk. It suggests that future studies can further explore the significance of these two variants in different populations.

*ERCC5* gene is a structure-specific endonuclease and 5′-3′ exonuclease (Scherly et al., 1993) that are required to participate in two NER subpathways and play a key role in carcinogenesis. *ERCC5* deficiency results in defective DNA repair and dysregulation of gene transcription (Koeppel et al., 2004). A previous study systematically estimated the association between *ERCC5* rs17655 variant and overall cancer risk showed that *ERCC5* rs17655 variant was not associated with cancer risk susceptibility (Zhu et al., 2012). In our meta-analysis, six more studies on CRC were included, and the results showed that rs17655 was significantly associated with increased risk of CRC in all five genetic models Furthermore, this association persisted in the Asian population. Although the detailed mechanism by which the SNPs in *ERCC5* are associated with CRC susceptibility is unclear, our findings could provide new insights into the genetic factors underlying cancer susceptibility and carcinogenesis.

In addition, *XPC* is the main damage sensor in GG-NER. It is involved in DNA damage recognition and DNA repair activation of NER mechanism, including UV-induced damage, in-chain crosslinking, and photoproducts (Christmann et al., 2003). Several significant SNPs have been identified in *XPC* loci, including rs2228000 and rs2228001. A meta-analysis by Wang et al. (2015) showed no evidence of a significant association between *XPC* variant and CRC risk, persisted in subgroup analyses of ethnicity and study design, which is consistent with our findings. Our subgroup analysis showed risk or protective significance in different populations, possibly due to relatively small sample size and the influence of different genetic backgrounds. Further studies of homogenous CRC patients and well-matched controls are necessary.

In this meta-analysis, to ensure comprehensive inclusion of the literature, keywords and their synonyms were included in the search. If the word appeared in the title or abstract or even the full text, it was initially included. Then, according to the inclusion criteria, a rigorous screening process was performed. The initial collection of 6,617 articles included many articles whose research topic was not related to CRC, NER, or SNP, as well as some for which the original study data were not available through the original text or by contacting the authors directly. In addition, some SNP studies of NER related genes with only one or two reports were also excluded in order to reduce bias. Finally, A total of 29 case-control studies were eligible. Moreover, to reduce the risk of drawing false positive or false negative conclusion, we performed TSA in this meta-analysis. TSA tests the confidence of the determined results by combining estimates of the size of the information with adjustment thresholds for statistical significance of the cumulative meta-analysis.

There are certain advantages that should be acknowledged. First, we included high-quality case-control studies with genotypes that met the Hardy-Weinberg balance. In addition, there was no significant publication bias in all comparisons, and the sensitivity analysis showed robustness. Nevertheless, there are some limitations to our study. Firstly, the present meta-analysis could not include several relevant studies owing to the lack of raw data, improper publication formats or publication limitations. Secondly, the study was based on unadjusted effect estimates and 95% CI, and lacked information such as age and gender, which may lead to confounding bias. Finally, our meta-analysis did not include environmental factors such as smoking, alcohol consumption or viral infections. It did not examine the effects of interactions between genes or genes and the environment.

## 5 Conclusion

In conclusion, our meta-analysis of studies in different populations indicated that *ERCC1* rs11615, *ERCC1* rs3212986, *ERCC2* rs1799793, and *ERCC5* rs17655 were significantly associated with CRC risk. Conversely, the results showed no significant association with CRC risk of four SNPs, including *ERCC2* rs13181, *ERCC4* rs1800067, and two SNPs of *XPC* (rs2228001 and rs2228000). In future research, the following aspects should be paid attention to. Firstly, there may be a need for further research on different SNP topics in the NER gene, involving broader population regions and sample sizes. Secondly, gene-environment interactions and gene-gene interactions should also be considered in subsequent studies. Finally, functional studies are required to elucidate the underlying mechanisms of NER genetic variants in tumorigenesis.

## Data Availability

The original contributions presented in the study are included in the article/Supplementary Material, further inquiries can be directed to the corresponding author.
