# Supplementary material for: Polymorphisms of nucleotide excision repair genes associated with colorectal cancer risk: Meta-analysis and trial sequential analysis
Source: Front Genet. 2022 Oct 31;13:1009938. doi: 10.3389/fgene.2022.1009938 (PMC9659581; doi:10.3389/fgene.2022.1009938)
Supplement: Supplementary file 1 [file DataSheet3.docx]

**Supplementary Information**

1. **Pubmed：**

(("colorectal"[Title/Abstract] OR "colonic"[Title/Abstract] OR "rectal"[Title/Abstract] OR "colon"[Title/Abstract] OR "rectum"[Title/Abstract]) AND ("cancer"[Title/Abstract] OR "carcinoma"[Title/Abstract] OR "neoplasms"[Title/Abstract] OR "tumor"[Title/Abstract]) AND ("single nucleotide polymorphism"[Title/Abstract] OR "Polymorphism"[Title/Abstract] OR "SNP"[Title/Abstract] OR "variant"[Title/Abstract] OR "variation"[Title/Abstract]) AND ("nucleotide excision repair"[Title/Abstract] OR "NER"[Title/Abstract] OR "dna repair"[Title/Abstract] OR "excision repair"[Title/Abstract])) AND (1000/1/1:2022/4/6[pdat])

1. **Web of science：**

(((TS=(colorectal OR colonic OR rectal OR colon OR rectum)) AND TS=(cancer OR carcinoma OR neoplasms OR tumor)) AND TS=(Single Nucleotide Polymorphism OR Polymorphism OR SNP OR variant OR variation)) AND TS=(nucleotide excision repair OR NER OR DNA repair OR excision repair)

1. **Embase：**

(((TS=(colorectal OR colonic OR rectal OR colon OR rectum)) AND TS=(cancer OR carcinoma OR neoplasms OR tumor)) AND TS=(Single Nucleotide Polymorphism OR Polymorphism OR SNP OR variant OR variation)) AND TS=(nucleotide excision repair OR NER OR DNA repair OR excision repair)

1. **Cochrane library:**

(colorectal OR colonic OR rectal OR colon R rectum) AND (cancer ORcarcinoma OR neoplasms OR tumor) AND (Single Nucleotide Polymorphism OR Polymorphism OR SNP OR variant OR variation) AND (nucleotide excision repair OR NER OR DNA repair OR excision repair)
