# Supplementary material for: Polymorphisms of nucleotide excision repair genes associated with colorectal cancer risk: Meta-analysis and trial sequential analysis
Source: Front Genet. 2022 Oct 31;13:1009938. doi: 10.3389/fgene.2022.1009938 (PMC9659581; doi:10.3389/fgene.2022.1009938)
Supplement: Supplementary file 2 [file DataSheet2.docx]

**Supplementary table 1. Quality assessment of studies included for meta-analysis**

| First author, year of publication [reference] | The Newcastle-Ottawa scale (NOS) for case-control study | | | | | | | | Total score |
| --- | --- | --- | --- | --- | --- | --- | --- | --- | --- |
|  | ① | ② | ③ | ④ | ⑤ | ⑥ | ⑦ | ⑧ |  |
| Mariana C Stern 2006 | ★ | ★ |  | ★ | ★★ |  | ★ |  | 6 |
| Victor Moreno 2006 | ★ | ★ | ★ | ★ | ★★ |  | ★ |  | 7 |
| C.Furu Skjelbred 2006 | ★ | ★ |  | ★ | ★★ |  | ★ |  | 6 |
| Chih-Ching Yeh 2007 | ★ | ★ |  | ★ | ★★ |  | ★ |  | 6 |
| B Pardini 2008 | ★ | ★ |  | ★ | ★★ |  | ★ |  | 6 |
| Tomasz Sliwinski 2009 | ★ | ★ |  | ★ | ★★ |  | ★ | ★ | 7 |
| Wang LiLi 2009 | ★ | ★ |  | ★ | ★★ |  | ★ |  | 6 |
| K Jelonek 2010 | ★ | ★ |  | ★ | ★★ |  | ★ |  | 6 |
| Ayse Basak Engin 2010 | ★ | ★ |  | ★ | ★★ |  | ★ |  | 6 |
| Jingwen Wang 2010 | ★ | ★ | ★ | ★ | ★★ |  | ★ |  | 7 |
| Emel Canbay 2011 | ★ | ★ | ★ | ★ | ★★ |  | ★ |  | 7 |
| Duo Liu 2012 | ★ | ★ |  | ★ | ★★ |  | ★ | ★ | 7 |
| Justyna Gil 2012 | ★ | ★ |  | ★ | ★★ |  | ★ |  | 6 |
| Susan E Steck 2014 | ★ | ★ | ★ | ★ | ★★ |  | ★ |  | 7 |
| Min Ni 2014 | ★ | ★ | ★ | ★ | ★★ |  | ★ |  | 7 |
| Ruizhi Hou 2014 | ★ | ★ | ★ | ★ | ★★ |  | ★ |  | 7 |
| Haina Du 2014 | ★ | ★ | ★ | ★ | ★★ |  | ★ |  | 7 |
| Kang Sun 2015 | ★ | ★ | ★ | ★ | ★★ |  | ★ | ★ | 8 |
| K.P-Szczur 2015 | ★ | ★ | ★ | ★ | ★★ |  | ★ |  | 7 |
| H. Yang 2015 | ★ | ★ |  | ★ | ★★ |  | ★ |  | 6 |
| B. GÓMEZ-DÍAZ 2015 | ★ | ★ |  | ★ | ★★ |  | ★ |  | 6 |
| Jacek Kabzinski 2015 | ★ | ★ |  | ★ | ★★ |  | ★ |  | 6 |
| Sha Zhang 2017 | ★ | ★ | ★ | ★ | ★★ |  | ★ |  | 7 |
| Te-Cheng Yueh 2017 | ★ | ★ | ★ | ★ | ★★ |  | ★ |  | 7 |
| Qianye Zhang 2018 | ★ | ★ | ★ | ★ | ★★ |  | ★ |  | 7 |
| Dexi Jin 2019 | ★ | ★ | ★ | ★ | ★★ |  | ★ |  | 7 |
| Jinsong Su 2019 | ★ | ★ |  | ★ | ★★ |  | ★ |  | 6 |
| Eda Balkan 2020 | ★ | ★ |  | ★ | ★★ |  | ★ |  | 6 |
| Yan-Ke Li 2020 | ★ | ★ |  | ★ | ★★ |  | ★ |  | 6 |

Note: ① Is the case definition adequate? ② Representativeness of the cases ③ Selection of Controls ④ Definition of Controls ⑤ Comparability of cases and controls on the basis of the design or analysis ⑥ Ascertainment of exposure ⑦ Same method of ascertainment for cases and controls ⑧ Non-Response rate

**Supplementary table 2. Genotyping and analysis results of the studied SNPs**

| Gene | SNP ID | Author | Year | Case | | | Control | | | Significant in genetic models |
| --- | --- | --- | --- | --- | --- | --- | --- | --- | --- | --- |
| ERCC1 | rs11615 |  |  | TT | TC | CC | TT | TC | CC | YES^b^ |
|  |  | Victor Moreno | 2006 | 64 | 138 | 132 | 52 | 126 | 123 |  |
|  |  | Ruizhi Hou | 2014 | 37 | 94 | 73 | 29 | 90 | 85 |  |
|  |  | Min Ni | 2014 | 14 | 82 | 117 | 19 | 86 | 135 |  |
|  |  | H. Yang | 2015 | 50 | 121 | 108 | 49 | 133 | 134 |  |
|  |  | B. GÓMEZ-DÍAZ | 2015 | 15 | 47 | 46 | 11 | 50 | 58 |  |
|  |  | Te-Cheng Yueh | 2017 | 71 | 131 | 160 | 43 | 139 | 180 |  |
|  |  | Yan-Ke Li | 2020 | 39 | 293 | 518 | 48 | 305 | 494 |  |
| ERCC1 | rs3212986 |  |  | CC | CA | AA | CC | CA | AA |  |
|  |  | Victor Moreno | 2006 | 217 | 105 | 27 | 178 | 102 | 20 | YES^a,b,d,e^ |
|  |  | Min Ni | 2014 | 104 | 91 | 18 | 142 | 88 | 10 |  |
|  |  | Ruizhi Hou | 2014 | 80 | 92 | 32 | 96 | 89 | 19 |  |
|  |  | Qianye Zhang | 2018 | 100 | 78 | 22 | 115 | 75 | 10 |  |
| ERCC2 | rs13181 |  |  | AA | AC | CC | AA | AC | CC | NO |
|  |  | Victor Moreno | 2006 | 158 | 150 | 49 | 135 | 145 | 38 |  |
|  |  | Camilla Furu Skjelbred | 2006 | 58 | 76 | 22 | 175 | 173 | 50 |  |
|  |  | Mariana C Stern | 2006 | 387 | 298 | 55 | 392 | 317 | 80 |  |
|  |  | Chih-Ching Yeh | 2007 | 602 | 112 | 3 | 631 | 96 | 4 |  |
|  |  | Wang LiLi | 2009 | 143 | 19 | 8 | 164 | 33 | 3 |  |
|  |  | Tomasz Sliwinski | 2009 | 56 | 33 | 11 | 42 | 41 | 17 |  |
|  |  | Jingwen Wang | 2010 | 138 | 130 | 34 | 137 | 117 | 37 |  |
|  |  | K Jelonek | 2010 | 54 | 47 | 22 | 66 | 68 | 19 |  |
|  |  | Emel Canbay | 2011 | 31 | 37 | 11 | 102 | 114 | 31 |  |
|  |  | Justyna Gil | 2012 | 46 | 72 | 15 | 32 | 46 | 22 |  |
|  |  | Susan E Steck group1 | 2014 | 114 | 136 | 49 | 209 | 251 | 65 |  |
|  |  | Susan E Steck group2 | 2014 | 128 | 83 | 13 | 186 | 115 | 19 |  |
|  |  | Min Ni | 2014 | 176 | 35 | 2 | 201 | 38 | 1 |  |
|  |  | K. P-Szczur | 2015 | 244 | 327 | 110 | 592 | 767 | 276 |  |
|  |  | B. GÓMEZ-DÍAZ | 2015 | 69 | 33 | 6 | 74 | 39 | 6 |  |
|  |  | Jacek Kabzinski | 2015 | 32 | 84 | 112 | 40 | 176 | 10 |  |
|  |  | Sha ZHANG | 2017 | 240 | 75 | 15 | 261 | 51 | 0 |  |
|  |  | Dexi Jin | 2019 | 5 | 56 | 164 | 0 | 33 | 167 |  |
|  |  | Eda Balkan | 2020 | 14 | 16 | 10 | 21 | 0 | 19 |  |
| ERCC2 | rs1799793 |  |  | GG | GA | AA | GG | GA | AA | YES^d,e,f^ |
|  |  | Victor Moreno | 2006 | 95 | 91 | 100 | 77 | 72 | 63 |  |
|  |  | K Jelonek | 2010 | 41 | 59 | 21 | 33 | 65 | 15 |  |
|  |  | Justyna Gil | 2012 | 47 | 68 | 18 | 32 | 55 | 11 |  |
|  |  | Susan E Steck | 2014 | 170 | 45 | 5 | 258 | 60 | 3 |  |
|  |  | Min Ni | 2014 | 182 | 26 | 5 | 210 | 27 | 3 |  |
|  |  | B. GÓMEZ-DÍAZ | 2015 | 74 | 26 | 8 | 81 | 23 | 15 |  |
|  |  | Jacek Kabzinski | 2015 | 91 | 120 | 20 | 109 | 118 | 2 |  |
| ERCC4 | rs1800067 |  |  | GG | GA | AA | GG | GA | AA | NO |
|  |  | Victor Moreno | 2006 | 282 | 71 | 7 | 257 | 61 | 5 |  |
|  |  | Justyna Gil | 2012 | 119 | 14 | 0 | 83 | 15 | 0 |  |
|  |  | Susan E Steck group1 | 2014 | 251 | 52 | 1 | 455 | 74 | 7 |  |
|  |  | Susan E Steck group2 | 2014 | 217 | 8 | 1 | 307 | 16 | 0 |  |
| ERCC5 | rs17655 |  |  | GG | GC | CC | GG | GC | CC | YES^a,b,c,d,f^ |
|  |  | B Pardini | 2008 | 334 | 177 | 21 | 356 | 153 | 23 |  |
|  |  | Emel Canbay | 2011 | 43 | 34 | 2 | 148 | 83 | 16 |  |
|  |  | Justyna Gil | 2012 | 86 | 35 | 11 | 64 | 31 | 5 |  |
|  |  | Duo Liu | 2012 | 233 | 603 | 192 | 329 | 537 | 219 |  |
|  |  | Haina Du | 2014 | 286 | 459 | 133 | 355 | 405 | 124 |  |
|  |  | Susan E Steck group1 | 2014 | 183 | 100 | 15 | 335 | 170 | 27 |  |
|  |  | Susan E Steck group2 | 2014 | 65 | 120 | 39 | 100 | 151 | 66 |  |
|  |  | Jacek Kabzinski | 2015 | 36 | 171 | 27 | 43 | 175 | 20 |  |
|  |  | Jinsong Su | 2019 | 248 | 510 | 261 | 265 | 515 | 256 |  |
| XPC | rs2228001 |  |  | GG | GT | TT | GG | GT | TT | NO |
|  |  | Ayse Basak Engin | 2010 | 25 | 63 | 22 | 36 | 55 | 25 |  |
|  |  | Justyna Gil | 2012 | 14 | 71 | 48 | 11 | 46 | 43 |  |
|  |  | Duo Liu | 2012 | 103 | 565 | 360 | 132 | 500 | 453 |  |
|  |  | Susan E Steck group1 | 2014 | 52 | 148 | 103 | 89 | 252 | 191 |  |
|  |  | Susan E Steck group2 | 2014 | 9 | 91 | 126 | 31 | 142 | 149 |  |
|  |  | K. P-Szczur | 2015 | 82 | 202 | 187 | 209 | 647 | 480 |  |
| XPC | rs2228000 |  |  | GG | GA | AA | GG | GA | AA | NO |
|  |  | Susan E Steck group1 | 2014 | 177 | 104 | 22 | 293 | 207 | 35 |  |
|  |  | Susan E Steck group2 | 2014 | 175 | 51 | 2 | 276 | 47 | 0 |  |
|  |  | K. P-Szczur | 2015 | 443 | 269 | 41 | 548 | 563 | 177 |  |
|  |  | Kang Sun | 2015 | 276 | 465 | 149 | 321 | 510 | 79 |  |

Note: ^a^ Allele model; ^b^ Homozygous model; ^c^ Heterozygous model; ^d^ Dominant model; ^e^ Recessive model; ^f^ Over-dominant model.
