# Supplementary material for: Polymorphisms of nucleotide excision repair genes associated with colorectal cancer risk: Meta-analysis and trial sequential analysis
Source: Front Genet. 2022 Oct 31;13:1009938. doi: 10.3389/fgene.2022.1009938 (PMC9659581; doi:10.3389/fgene.2022.1009938)
Supplement: Supplementary file 3 [file DataSheet1.docx]

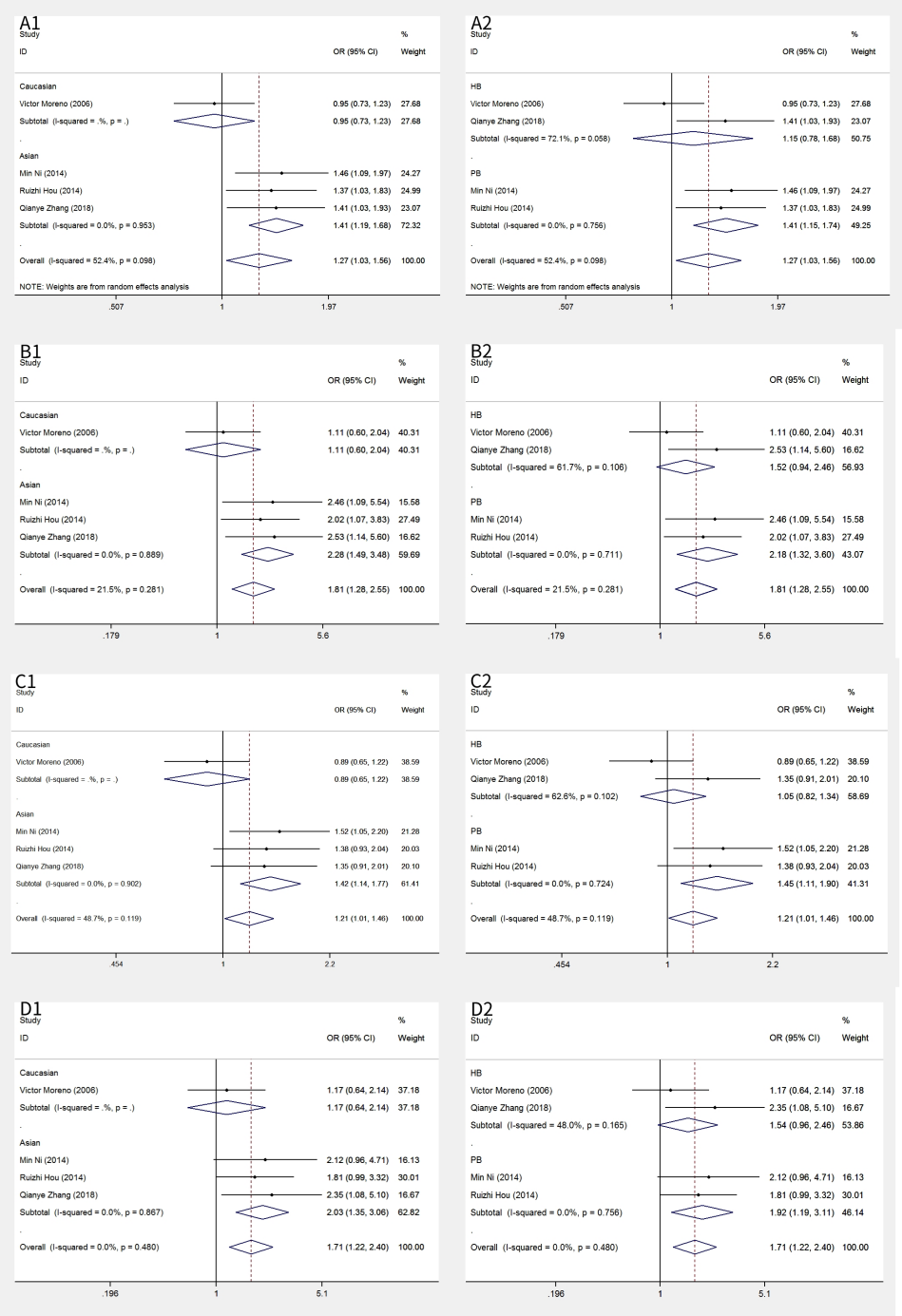


Supplement figure S1. Subgroup forest plot related to ERCC1 rs3212986 and risk of CRC. (A) Allele model (B) Homozygous model (C) Dominant model (D) Recessive model (1) Ethnicity subgroup (2) Source of control subgroup


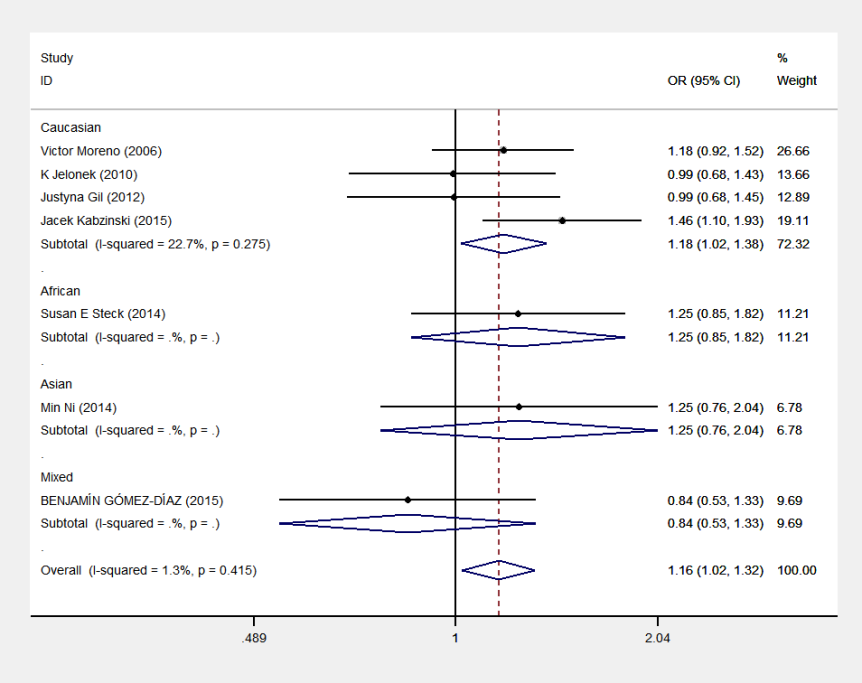


Supplement figure S2. Ethnicity subgroup forest plot related to ERCC2 rs1799793 and risk of CRC in the allele model.


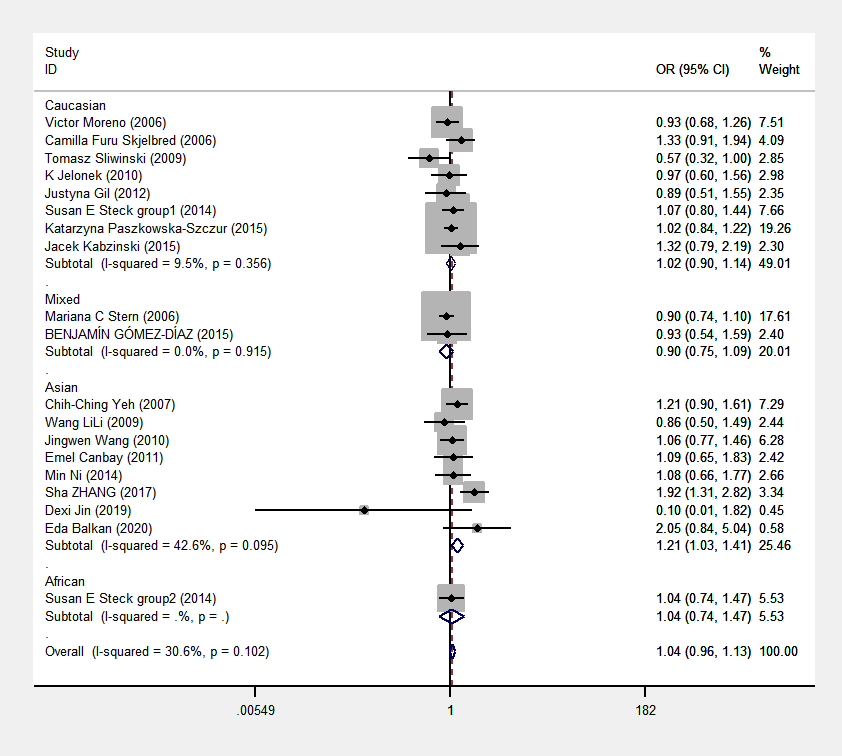


Supplement figure S3. Ethnicity subgroup forest plot related to ERCC2 rs13181 and risk of CRC in the dominant model.


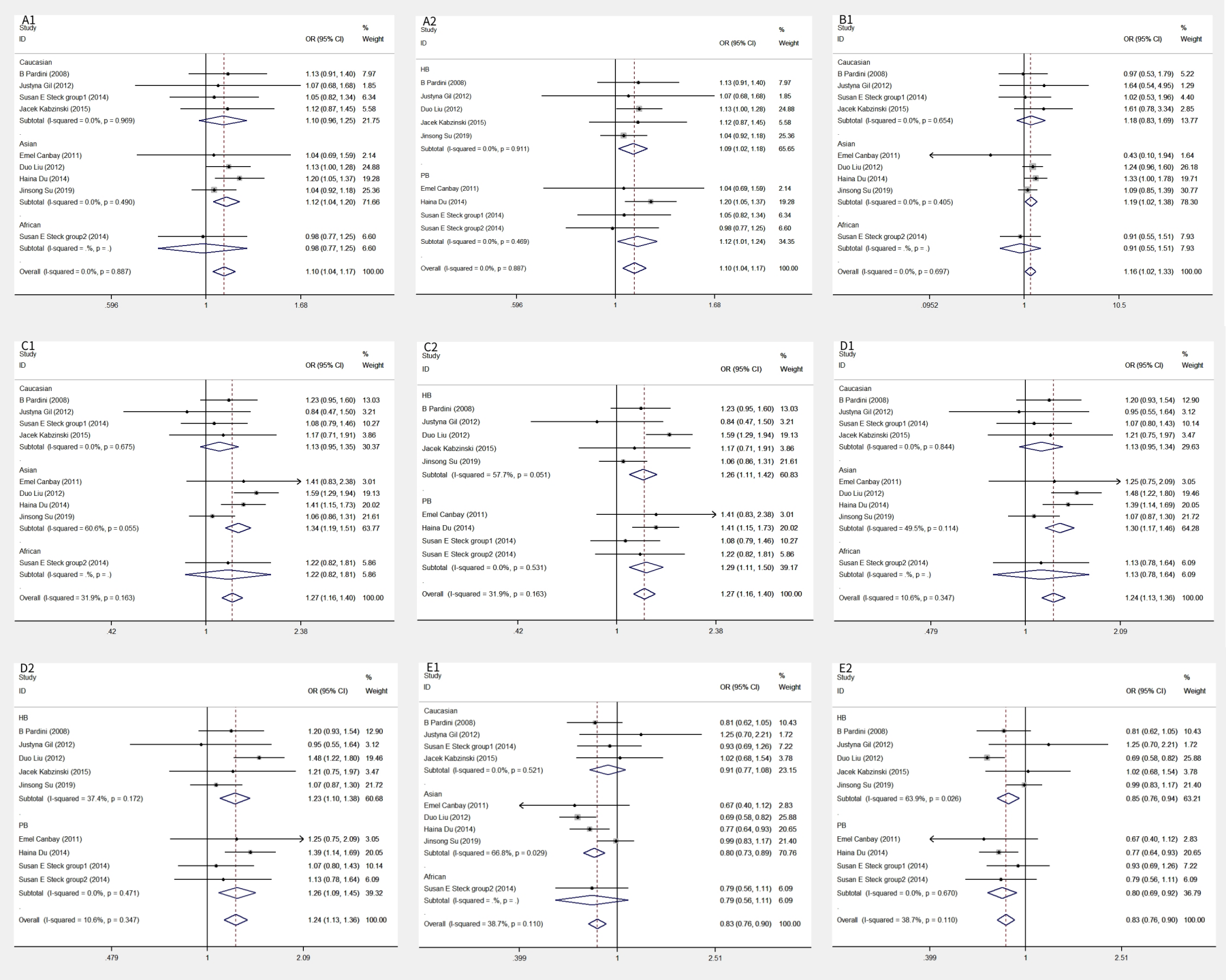


Supplement figure S4. Subgroup forest plot related to ERCC5 rs17655 and risk of CRC. (A) Allele model (B) Homozygous model (C) Heterozygous model (D) Dominant model (E) Over-dominant model (1) Ethnicity subgroup (2) Source of control subgroup


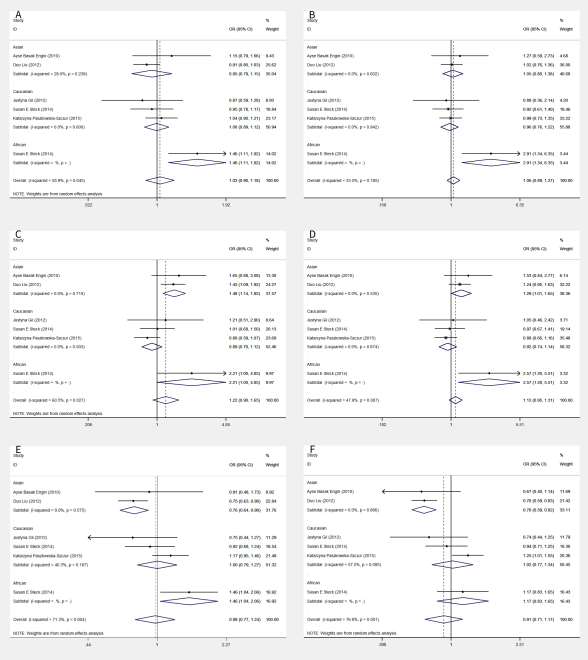


Supplement figure S5. Ethnicity subgroup forest plot related to XPC rs2228001 and risk of CRC. (A) Allele model (B) Homozygous model (C) Heterozygous model (D) Dominant model (E) Recessive model (F) Over-dominant model


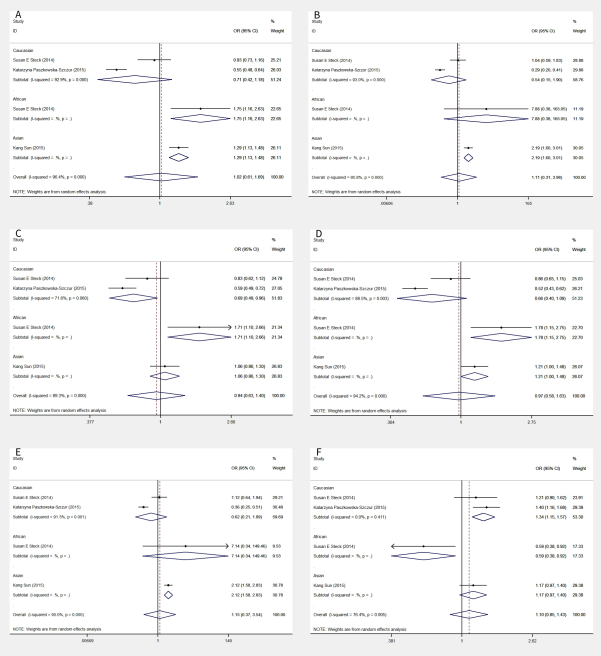


Supplement figure S6. Ethnicity subgroup forest plot related to XPC rs2228000 and risk of CRC. (A) Allele model (B) Homozygous model (C) Heterozygous model (D) Dominant model (E) Recessive model (F) Over-dominant model


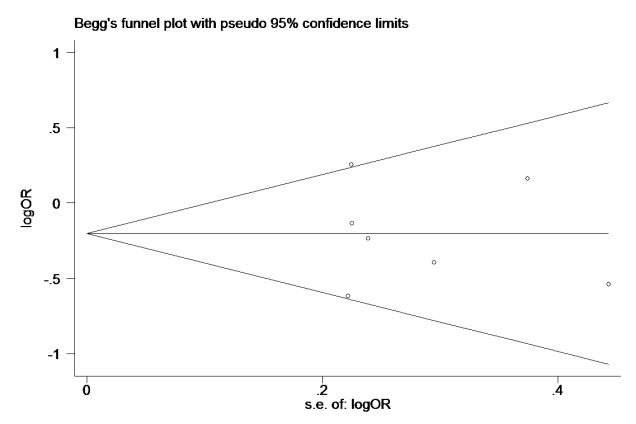

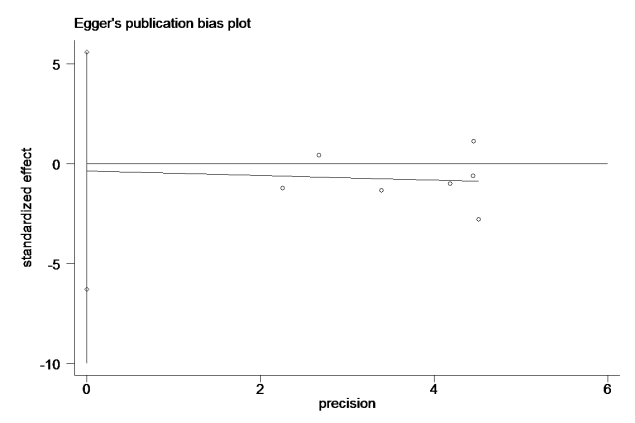

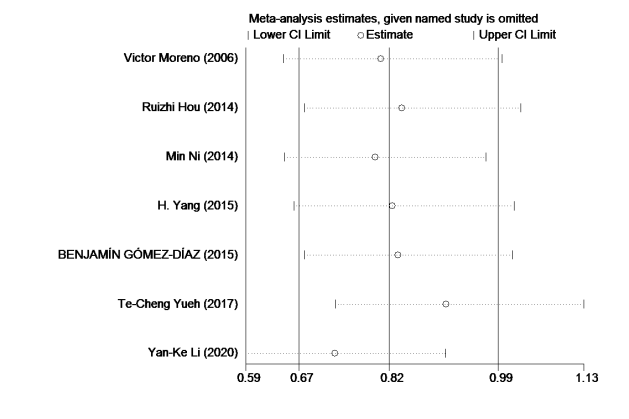


Supplement figure S7. Begg’s and Egger’s funnel plot for publication bias test and sensitivity analysis related to *ERCC1* rs11615 and risk of CRC in the homozygous model.

A.


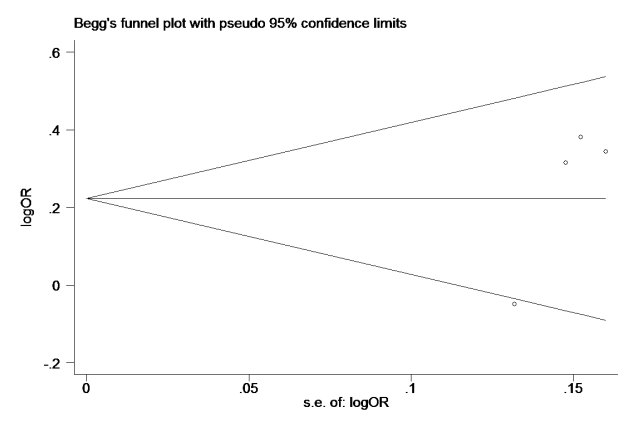

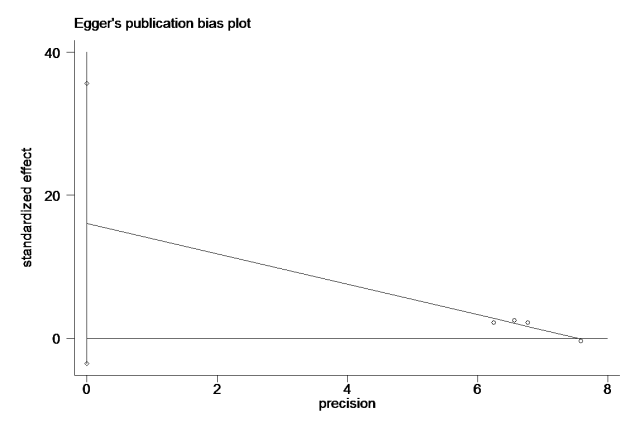

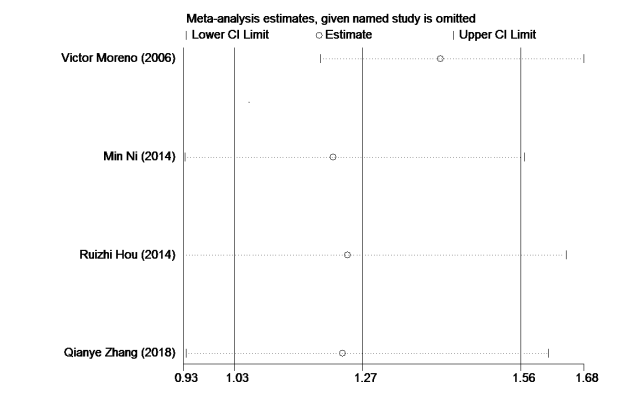


B.


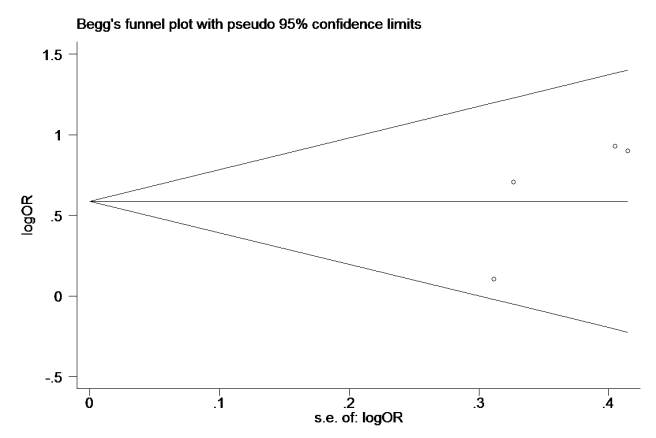

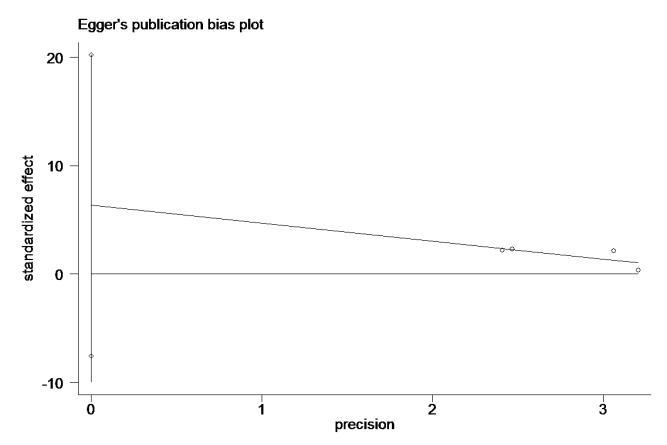

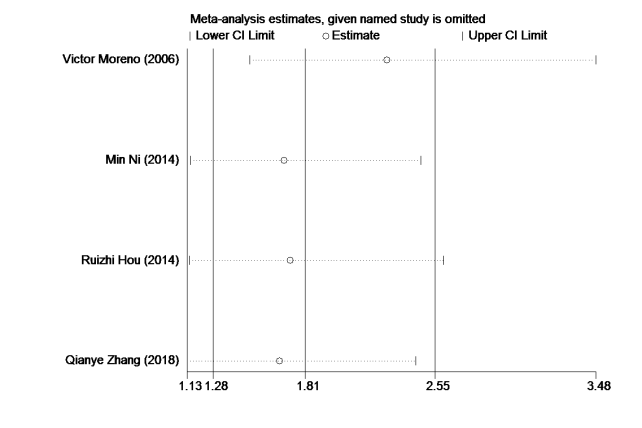


C.


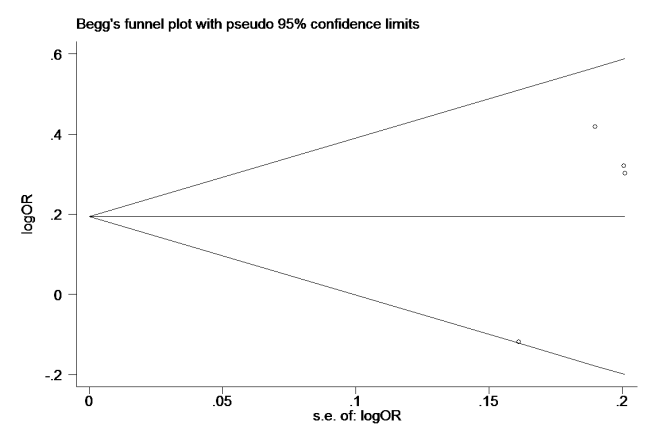

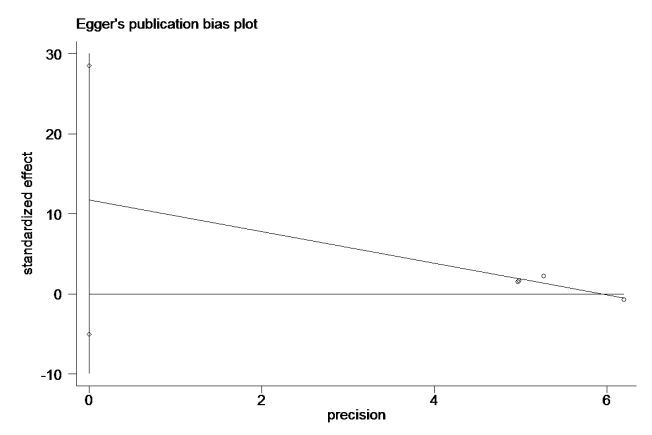

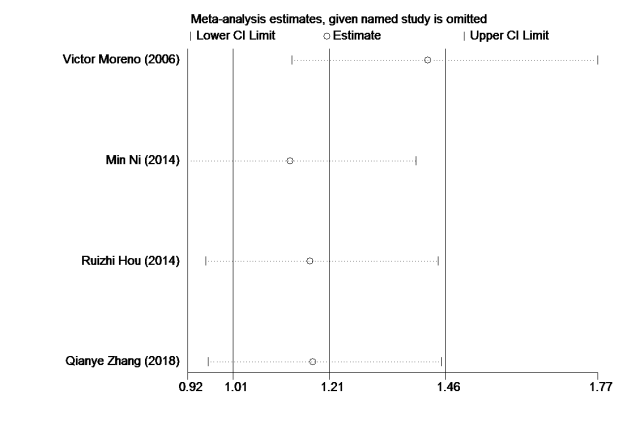


D.


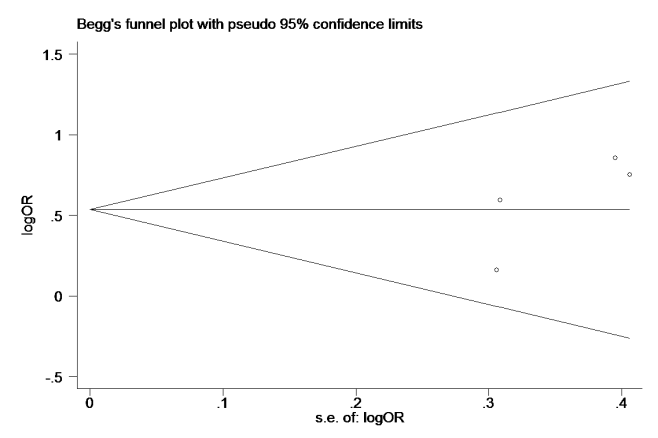

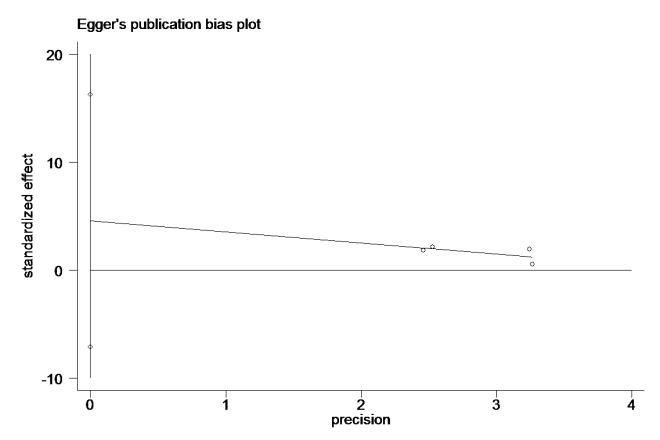

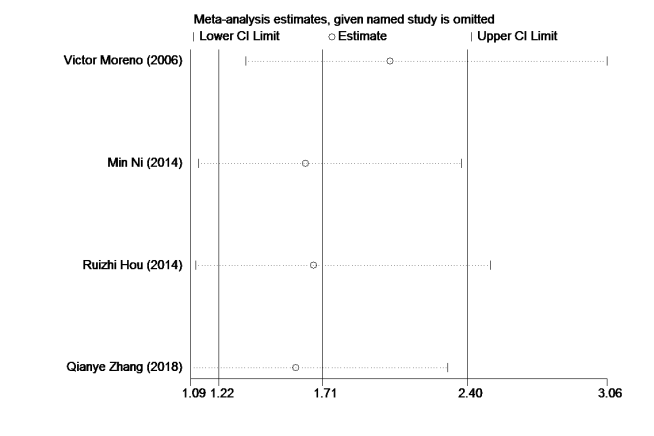


Supplement figure S8. Begg’s and Egger’s funnel plot for publication bias test and sensitivity analysis related to *ERCC1* rs3212986 and risk of CRC. (A) Allele model (B) Homozygous model (C) Dominant model (D) Recessive model


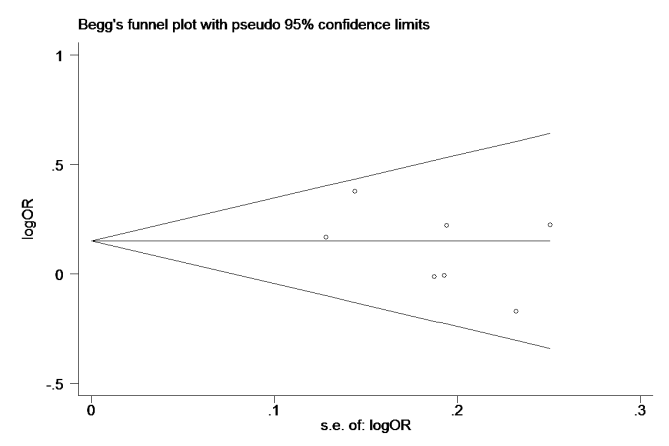

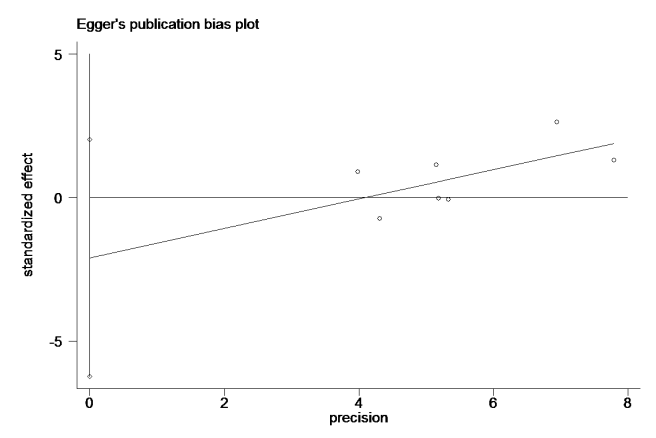

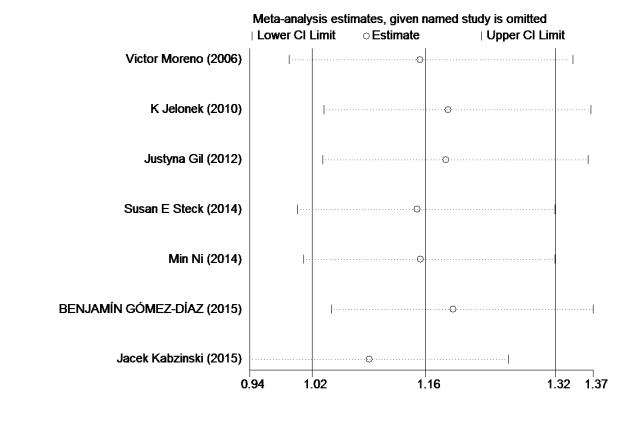


Supplement figure S9. Begg’s and Egger’s funnel plot for publication bias test and sensitivity analysis related to *ERCC2* rs1799793 and risk of CRC in the allele model.

A.


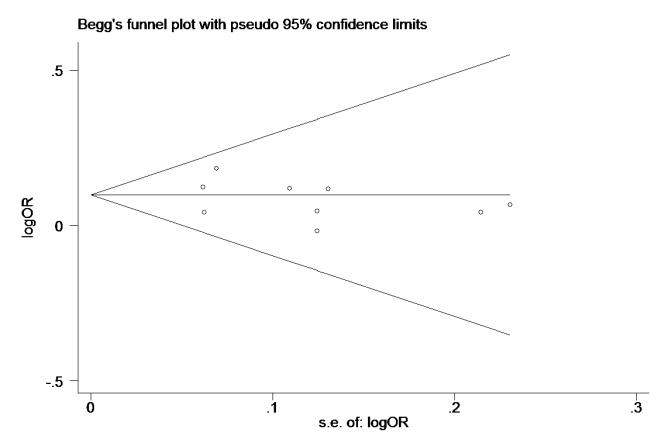

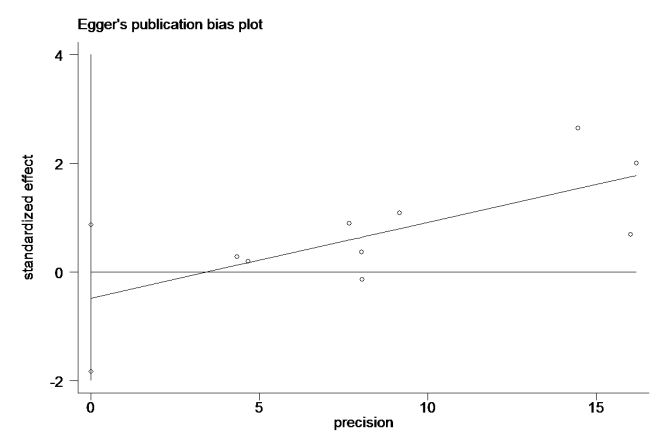

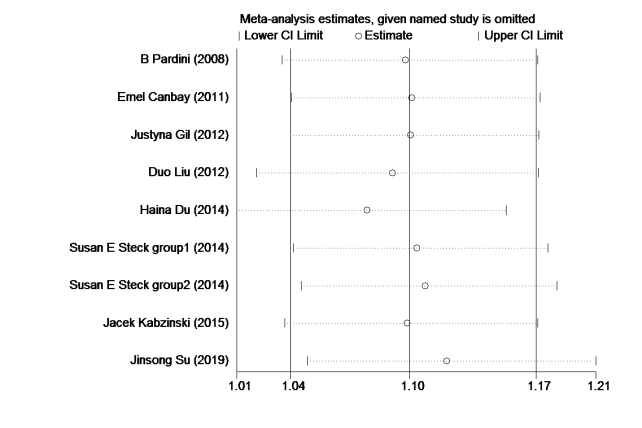


B.


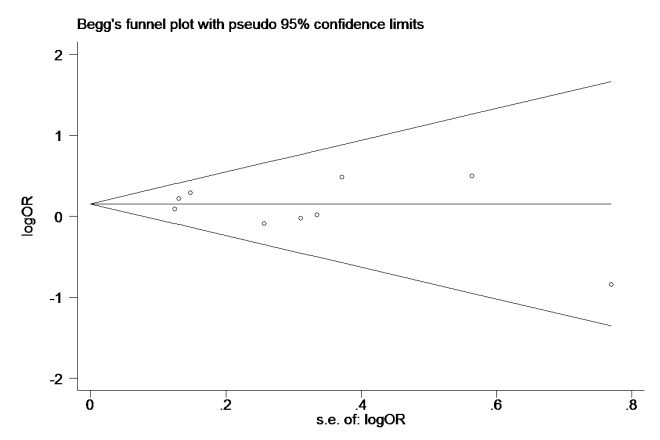

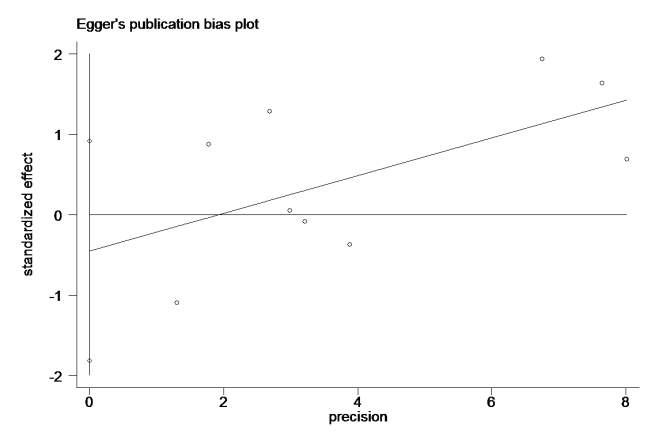

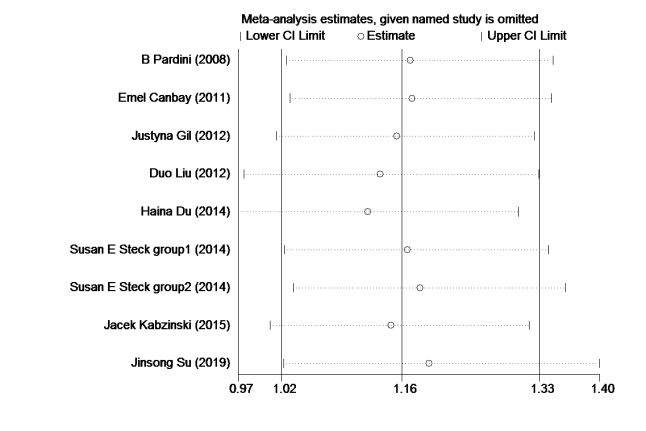


C.


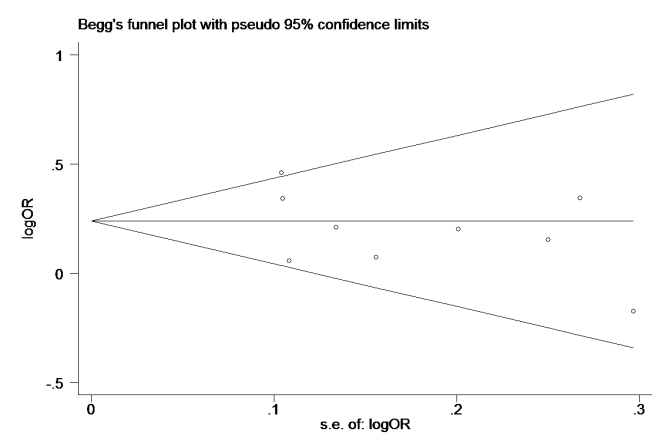

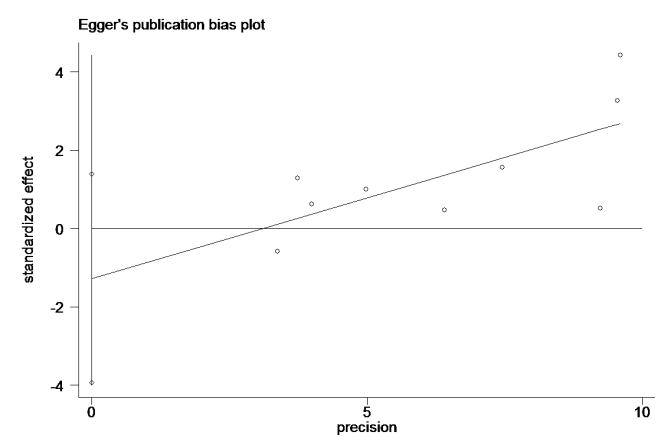

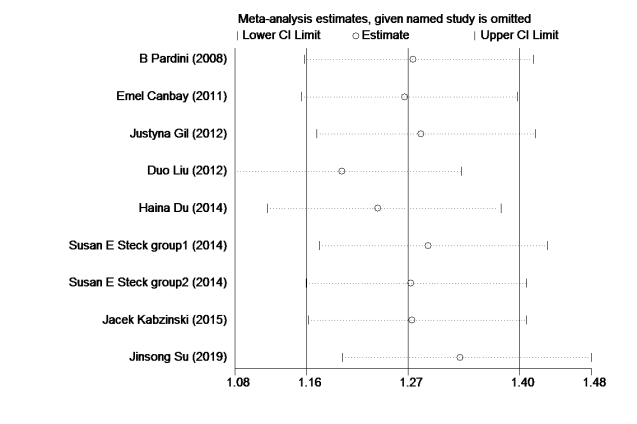


D.


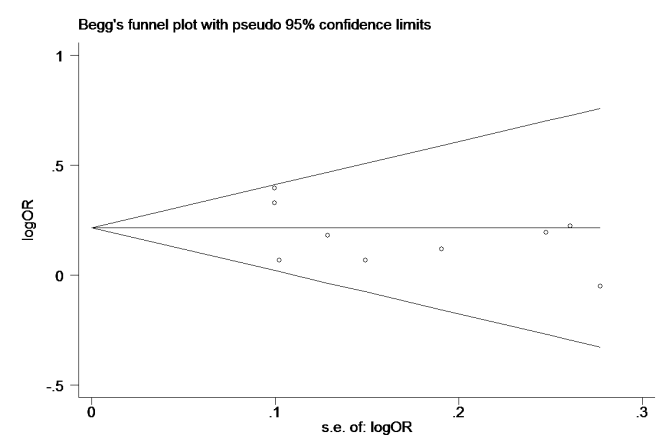

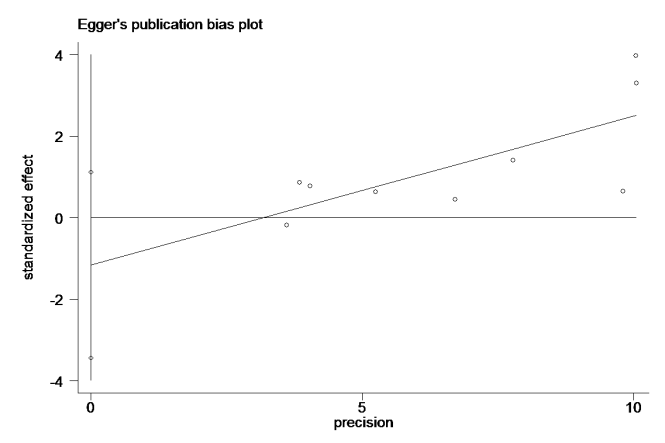

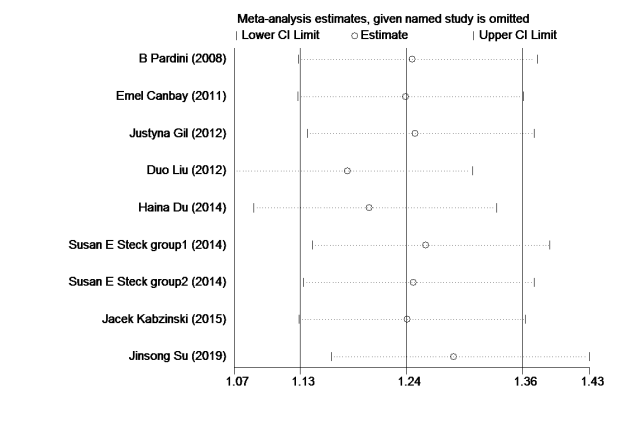


E.


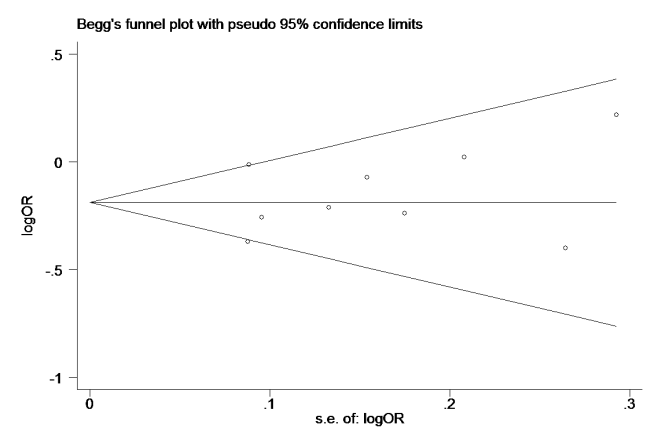

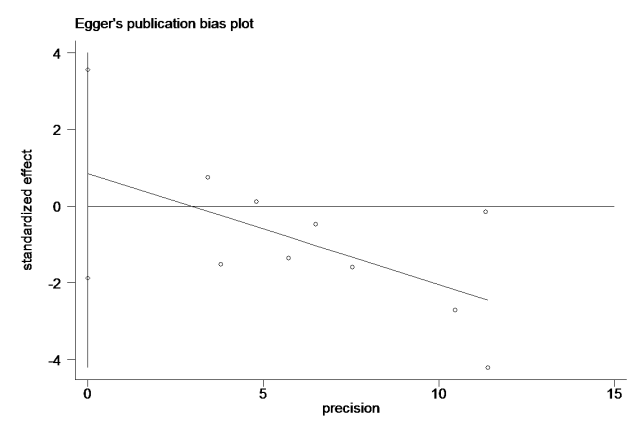

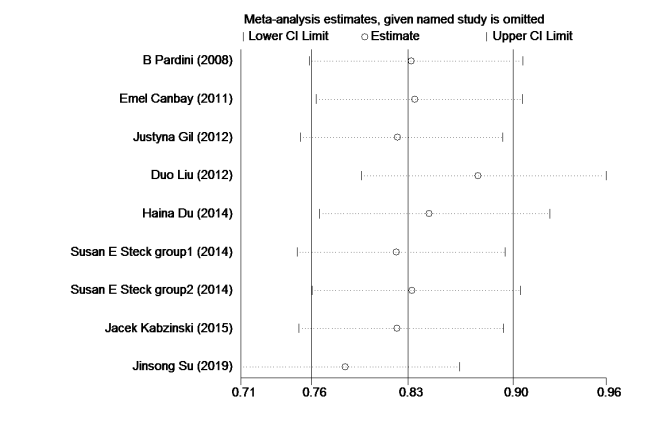


Supplement figure S10. Begg’s and Egger’s funnel plot for publication bias test and sensitivity analysis related to *ERCC5* rs17655 and risk of CRC. (A) Allele model (B) Homozygous model (C) Heterozygous model (D) Dominant model (E) Over-dominant model
